# Nature-based interventions for enhancing resilience in children: a systematic review and meta-analysis

**DOI:** 10.1007/s44192-025-00258-7

**Published:** 2025-07-23

**Authors:** Myrian Sze Nga Fan, William Ho Cheung Li, Laurie Long Kwan Ho, Kai Chow Choi, Lophina Phiri, Sara Pacchiani, Brigitta Antal, Clement Shek Kei Cheung, Daoud Kamal Abu Khaleel

**Affiliations:** 1 The Chinese University of Hong Kong, Rm 831, Esther Lee Building, Hong Kong, China; 2 Universita Degli Studi Di Milano-Bicocca, Milan, Italy; 3 Károli Gáspár University of the Reformed Church in Hungary, Budapest, Hungary; 4 University of New South Wales, Sydney, Australia; 5 Hong Kong Institute of Vocational Education, Hong Kong, China

**Keywords:** Children, Mental health, Resilience, Nature-based interventions

## Abstract

Despite increasing interest in nature-based interventions (NBIs) for mental health, no or very few prior reviews have quantitatively synthesised their effects on children’s resilience, a key developmental outcome. This study systematically reviews and meta-analyses available evidence following PRISMA guideline. The Cumulative Index to Nursing and Allied Health Literature, Cochrane, Embase, Education Resources Information Center, Medline, APA PsycArticles, SPORTDiscus, and Web of Science were searched from inception to November 28, 2024. For additional studies, Google Scholar and references from included studies were searched. The review included randomised clinical trials, controlled trials, and single-group pre-post studies reporting resilience. Findings not synthesised quantitatively were summarised narratively. A total of 13 articles involved 15 studies with 2,571 participants (mean age 15.57 years; range 13 to 17 years; 1315 [53%] female). Random-effect meta-analysis indicated significant moderate-to-large short-term effects of NBIs on children’s resilience (standardised mean difference, 0.64; 95% confidence level, 0.36 to 0.91; *p* <.001). Substantial heterogeneity of intervention effects was observed (I^2^ = 98%). This review provides preliminary evidence that NBIs may be associated with short-term improvements in resilience among children, though results should be interpreted cautiously due to study limitations. This early evidence highlights a community-based approach that may support resilience development through nature. Sailing showed some promise as an early observation noted in subgroup analysis and narrative synthesis emphasising nature-human kinship. High-quality trials are needed to evaluate the effects of sailing on resilience and address gaps: the long-term effect of NBIs and their impact on children outside the 13–17 age range.

*Trail registration*: PROSPERO database (CRD42025634371).

**Supplementary Information:**

The online version contains supplementary material available at 10.1007/s44192-025-00258-7.

## Introduction

Adversity is a natural and inevitable part of life, affecting individuals from childhood through adulthood [1]. The ability to cope with adversity is pivotal in determining long-term mental health outcomes [2], as struggles in managing it can negatively impact adult developmental milestones [3]. In this regard, resilience emerges as a vital construct, empowering individuals to navigate life’s challenges while enhancing their functionality and quality of life [4]. Resilience is defined as the ability to bounce back from difficult experiences [5, 6]. It is characterised by mental, emotional, and behavioural flexibility in response to difficult life experiences [7]. Recent research has highlighted its role in health promotion and the mitigation of mental health problems [8, 9].

Resilience can be developed at any age, but the school-aged period offers a critical window of opportunity due to heightened brain plasticity during this stage of development [10]. Given that mental disorders often onset in late childhood and early adolescence [11], prioritising early resilience interventions becomes essential. This proactive approach aligned with a paradigm shift in mental health research from focusing on risk and psychopathology to the advocacy of resilience enhancement [12].

Recognising the effectiveness of a community-based approach in promoting mental health [13], the literature highlights the critical role of social support within the community to foster resilience [14], particularly with nonparental adults during childhood [15]. Leveraging support outside the healthcare system through community engagement and the involvement of community-based organisations cultivates a supportive environment [16]. While existing evidence identifies cognitive-behavioural theory as the most frequently reported approach for enhancing resilience among children [17], its application is largely confined to school settings [18], highlighting a gap in community-based initiatives.

Despite increasing interest in nature-based interventions (NBIs) to support mental health, no or very few prior systematic reviews have quantitatively synthesised their effects on resilience in children. NBIs provide a promising approach to enhance resilience by combining engagement with nature [19, 20] and community-based social elements [21]. NBIs are professionally guided programmes that involve structured, nature-based activities to promote health [22]. They incorporate three components: (1) place-based elements, (2) active bodily engagement, and (3) recognition of nature-human kinship [23]. The availability and quality of social resources are critical determinants in fostering resilience [7]. NBIs are designed to create inclusive and supportive environments that provide opportunities for individuals to engage, connect, and thrive, with intentionally structured settings with fewer demands and less stringent expectations than traditional ones [24]. This approach aligns with resilience as a strength-based concept, which focuses on building an individual’s capabilities and resources to bounce back rather than focusing on the deficits [25]. While prior systematic reviews [26, 27] broadly examined NBIs among youths, they differ significantly from the present study in both scope and methodology. Obeng et al. [26] conducted a narrative review focused on NBIs for young people in precarious situations, using a social work lens and spanning a broad age range. Roberts et al. [27] reviewed nature activities in relation to wellbeing but did not isolate resilience as a primary outcome and included children through young adults. Neither review conducted a meta-analysis. In contrast, the present study provides the first quantitative synthesis focused specifically on resilience outcomes in children under 18, thereby addressing a key gap in the NBI evidence base.

### Aim and objectives

This systematic review and meta-analysis aim to synthesise existing evidence on the effects of NBIs on children’s resilience. This review has two objectives: (1) to examine NBIs’ effects on children’s resilience and (2) to identify the core content used in delivery.

## Methods

This systematic review and meta-analysis were conducted and reported following the Preferred Reporting Items for Systematic Reviews and Meta-Analyses (PRISMA) guidelines. The review protocol is registered in the PROSPERO database (Identification number: CRD42025634371).

### Eligibility criteria

This review included all NBIs that reported resilience as an outcome measure in at least one of the primary or secondary outcomes outlined in the data collection items session for children aged 18 years or younger. In this review, NBIs were defined as interventions conducted in natural outdoor settings (e.g. forests, oceans, wilderness areas) and involving direct interaction with nature. Interventions occurring outdoors but lacking ecological immersion, such as urban schoolyard activities or artificial playground settings, were excluded. For instance, two studies involving structured sports in fenced outdoor courts were reviewed but excluded due to absence of a natural environment component. We considered a range of study designs, including randomised controlled trials (RCTs), controlled trials, and single-group pre-post studies. Detailed Population, Intervention, Control, and Outcomes (PICO) data are provided in eTable 1 of Appendix.

### Study selection and data extraction

We conducted a comprehensive search in Eight electronic bibliographic English databases, Cumulative Index to Nursing and Allied Health Literature (CICAHL), Cochrane Central Register of Controlled Trials (CENTRAL), Embase, Education Resources Information Center (ERIC), Medline, APA PsycArticles, SPORTDiscus, and Web of Science, from inception to November 28, 2024. The search strategies, including specific search terms and results, are detailed in eTables 2 to 10 of Appendix. No restrictions were placed on the publication year, and only peer-reviewed articles in English were included. Additionally, Google Scholar (Alphabet) and the references from the identified articles were manually checked for further potential studies. All records obtained through electronic searches were imported into EndNote version 20 (Clarivate). Duplicates were eliminated after exporting all records to a review management platform (Covidence) for screening and review.

Titles and abstracts were screened for eligibility criteria by two independent reviewers (M.S.N.F. and S.P.), followed by a thorough assessment of the full texts. A third reviewer (L.P.) was consulted on instances of differing opinions. Data were independently extracted from eligible studies by the same two reviewers using a shared extraction form, with a third reviewer involved in cases of disagreement. To assess the outcomes of NBIs, raw means and standard deviations (SDs), confidence intervals (CIs), t-values and sample sizes, as appropriate, were collected for both intervention and control groups to calculate standardised mean differences (SMDs) and their associated standard errors (SEs). Data was gathered and analysed from each study at the immediate post-intervention and follow-up evaluation time points.

### Risk of bias assessment

The methodological quality was assessed independently by two reviewers (M.S.N.F. and S.P.) using the revised Cochrane Risk-of-Bias tool for Randomized Trials (RoB 2) [28] and the Risk of Bias in Non-randomized Studies–of Interventions (ROBINS-I) [29]. The ROB 2 tool was employed to critically evaluate the quality of the included RCTs regarding randomness, concealment of participant allocation, blinding of participants and outcome assessors, the proportion of missing data, selective reporting, and other potential biases. The overall risk of bias was classified as low risk, some concerns, or high risk. For the nonrandomised studies, the ROBINS-I tool was utilised to assess the quality concerning selection bias, confounding, and measurement bias, with the overall risk of bias judgment, categorised as low risk, moderate risk, serious risk, or critical risk. In case of disagreement, a third reviewer (L.P.) was consulted.

### Data synthesis

If two or more clinically similar controlled trials, in terms of population and intervention, were available for each outcome, their results were pooled by meta-analysis. Effect estimates for the continuous outcome of resilience were pooled as SMD together with its 95% CI. The SMDs and their associated SEs of the individual studies were first calculated based on their reporting data, such as means, SDs, CIs, t-values, and sample sizes. A random-effects model using generic inverse variance method was applied for meta-analysis of the individual studies’ SMD and their SEs. The SMDs and their SEs for both the 2-arm parallel and single-group designs were calculated using the methods specified in Hedges and Olkin (1985) [30]. A conservative pre-post correlation coefficient, of 0.75 [31] was assumed for those single-group studies. The heterogeneity of intervention effects among studies was assessed using the I^2^ statistic, with low variation defined as I^2^ = 25%, moderate as I^2^ = 50%, and high as I^2^ = 75% [32]. The effect size (SMD) could be interpreted as small (SMD of 0.2), moderate (SMD of 0.5), and large (SMD of 0.8) [33]. Subgroup analysis was conducted to explore the differences in effect sizes across different type of NBIs. Sensitivity analyses were also conducted to examine the robustness of the meta-analysis results by (1) assuming a less conservative autocorrelation, r = 0.5, (2) excluding the single group studies, and (3) excluding those studies with serious or critical risk of bias. Furthermore, a supplementary analysis was conducted to pool the SMDs of the controlled trails. These estimates were based on within-intervention group pre-post changes with all single-group studies, to evaluate the within-group effect size and compare it with the pooled between-group effect size from the controlled trails. The meta-analyses were performed using Review Manager software version 5.4.1 (Cochrane Collaboration). Study authors were contacted to clarify missing data. Findings from studies that were not comparable and could not be included in the statistical pooling were summarized narratively. Subgroup analyses and interaction tests were conducted for groups with at least two studies. Data analysis was conducted from December 2 to December 6, 2024.

### Grading the evidence

Two reviewers (M.S.N.F. and L.P.) assessed the certainty of evidence using the Grading of Recommendations Assessment, Development, and Evaluation Guideline Development Tool (GRADEpro GDT) [34], rating it as moderate. Comprehensive details regarding the domains of the GRADE tool can be found in eTable 11 of Appendix. Publication bias was assessed using visual inspection of the funnel plot for asymmetry and quantitatively evaluated with the Egger’s statistical test. The funnel plot (eFigure 1 of Appendix) appeared symmetrical, and the Egger’s test yielded a *p*-value of 0.675, suggesting no significant evidence of publication bias.

## Results

### Systematic review

**Fig. 1:**
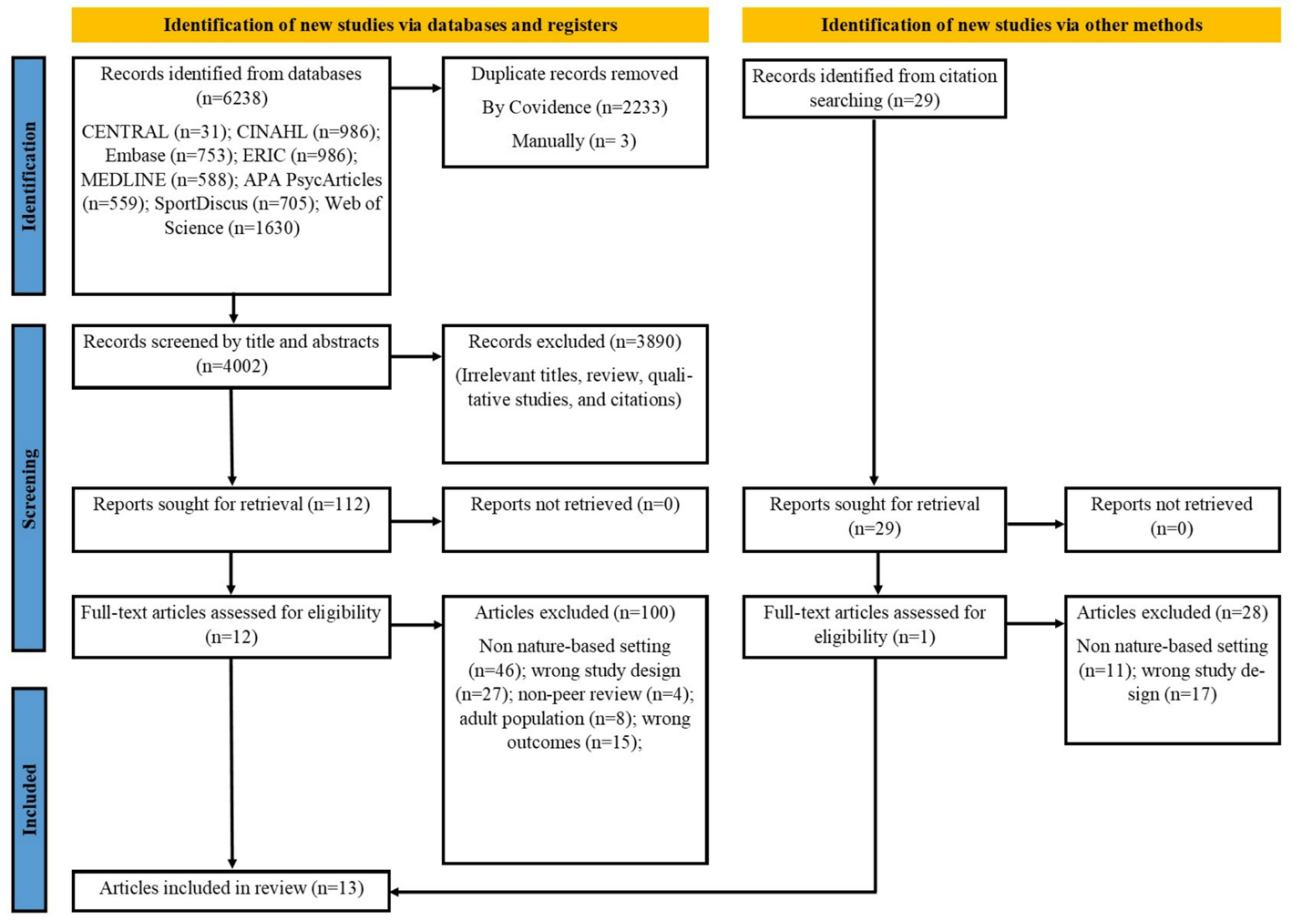
Flowchart for study retrieval and selection

A total of 13 articles [35–47] involving 2,571 participants (mean age 15.57 years; range 13 to 17 years; 1315 females [53%]) and comprised 15 studies, as two articles both reported two studies [43, 45]. The flowchart for study retrieval and selection is presented in Fig. 1. In total, 6,238 records in English were retrieved from which 2,236 duplicates were excluded. The titles and abstracts of the remaining 4,002 records were screened, and 112 were retained for full-text review, of which 100 articles were excluded. After assessing eligibility, 12 articles were included, along with one additional article identified through citation searching, resulting in a total of 13 articles [35–47].

#### Characteristics of the included studies

The 15 studies included one RCT [38], three controlled trials [41, 43, 47], and 11 single-group pre-post studies [35–39, 42–46]. The included studies were published in English within the last 15 years (2009–2024). Seven studies were conducted in New Zealand [37, 41, 43, 45, 47], two in Australia [39, 42], and the remaining studies were from Botswana [44], Canada [46], Italy [38], Hong Kong [40], the United Kingdom [36], and the United States [35]. The sample size ranged from 19 [42] to 650 [44]. The attrition rate ranged from 0 to 48.4%. Only two studies [39, 40] mentioned the use of standardized protocols to ensure fidelity and only one study [40] indicated the use of periodic checks to ensure adherence to the protocol. None of the studies reported mean fidelity ratings with numeric data. The characteristics of the included studies are outlined in eTable 12 of Appendix.

#### Characteristics of NBIs

The characteristics of NBIs varied across studies and are detailed in Table 1. All studies incorporated adventure-based experiential learning and were delivered in a group-based format. Four studies focused on wilderness experience [36, 41, 42, 44]. Eleven studies incorporated recreational activities such as sailing [37, 38, 43, 45, 47], canoeing [46], hiking, abseiling, rock climbing, or combinations of these [35, 39, 40]. All included studies were categorised based on components of intentional therapeutic processes within outdoor therapies [23]. All involved active bodily engagement with nature. Of the 15 studies, ten recognised nature-human kinship [36–38, 43–47]. Seven focused on sailing [37, 38, 43, 45, 47], one on canoeing [46], and two on wilderness experience [36, 44]. Qualified instructors are identified as the most common facilitators. Among the five studies incorporating an interdisciplinary approach [38, 40, 43, 44], one was led by nurses and qualified instructors [40], one by researchers and qualified instructors [38], one by a social worker and psychologist [44], and two by psychologists and qualified instructors [43]. Data on funding sources and socioeconomic status were inconsistently reported. Eleven studies [35–39, 41–43, 45] did not disclose funding status, while four [40, 44, 46, 47] reported it. Only two studies provided socioeconomic information [37, 40]. As a result, a direct association between funding and barriers to access cannot be established based on the available data.

**Table 1:** Summary description of the intervention of included studies

Authors	NBI component	Intervention								Control
		Content	Delivery mode	Teaching and learning modality	Program duration (week)	Session frequency (per week)	Session length (minutes)	Intervenors	Format	
Albedry et al. [35]	(1)(2)	Recreational activity: Hiking, rock climbing, camping	Face-to-face	Adventure-based experiential learning	15	2–3 times	90	Qualified instructors	Group-based	NA
Allan et al. [36]	(1)(2)(3)	Wilderness experience	Face-to-face	Adventure-based experiential learning	Five days			Researchers	Group-based	NA
Arahanga-Doyle et al. [37]	(1)(2)(3)	Recreational activity: sailing	Face-to-face	Adventure-based experiential learning	Seven days			Qualified instructors	Group-based	NA
Boarini et al. [38]	(1)(2)(3)	Recreational activity: sailing	Face-to-face	Adventure-based experiential learning	Five days			Qualified instructors and researchers	Group-based	NA
Bowen et al. [39]	(1)(2)	Recreational activity: Bushwalking, abseiling, cross-country skiing, hiking, rock climbing, caving, rafting	Face-to-face	Adventure-based experiential learning	10	1	nd	Practitioners	Group-based	NA
Chung et al. [40]	(1)(2)	Recreational activity: Abseiling, hiking	Face-to-face	Adventure-based experiential learning	Two days			Nurses and qualified instructors	Group-based	Leisure activity
Furness et al. [41]	(1)(2)	Wilderness experience	Face-to-face	Adventure-based experiential learning	Two to three weeks			nd	Group-based	No intervention
Gillespie and Allen-Craig [42]	(1)(2)	Wilderness experience	Face-to-face	Adventure-based experiential learning	5	1	120	nd	Group-based	NA
Hayhurst et al. [43]_Study 1	(1)(2)(3)	Recreational activity: sailing	Face-to-face	Adventure-based experiential learning	Ten days			Psychologist and qualified instructors	Group-based	Psychology course
Hayhurst et al. [43]_Study 2	(1)(2)(3)	Recreational activity: sailing	Face-to-face	Adventure-based experiential learning	Ten days			Psychologist and qualified instructors	Group-based	NA
Katisi et al. [44]	(1)(2)(3)	Wilderness experience	Face-to-face	Adventure-based experiential learning	Two weeks			Social worker and psychologist	Group-based	NA
Koni et al. [45]_Study 1	(1)(2)(3)	Recreational activity: sailing	Face-to-face	Adventure-based experiential learning	Ten days			Sailing crew	Group-based	NA
Koni et al. [45]_Study 2	(1)(2)(3)	Recreational activity: sailing	Face-to-face	Adventure-based experiential learning	Seven days			Sailing crew	Group-based	NA
Ritchie et al. [46]	(1)(2)(3)	Recreational activity: canoeing	Face-to-face	Adventure-based experiential learning	Ten days			Health leaders	Group-based	NA
Scarf et al. [47]	(1)(2)(3)	Recreational activity: sailing	Face-to-face	Adventure-based experiential learning	Ten days			Qualified instructors	Group-based	No intervention

^1^NBI component adopted from the intentional therapeutic processes under outdoor therapies: (1)—place-based, (2)—feature active bodily engagement, (3) recognise nature-human kinship

^2^nd, No data

#### Risk of bias

One RCT [40] demonstrated a low risk of bias (ROB) (eFigure 2 of Appendix). Among the 14 non-RCT studies, ten were classified as having a moderate overall ROB [37, 38, 41–45, 47]. One had a serious ROB [36]. Three were deemed to have a critical overall ROB [35, 39, 46] (eFigure 3 of Appendix).

### Meta-analyses of resilience

**Fig. 2:**
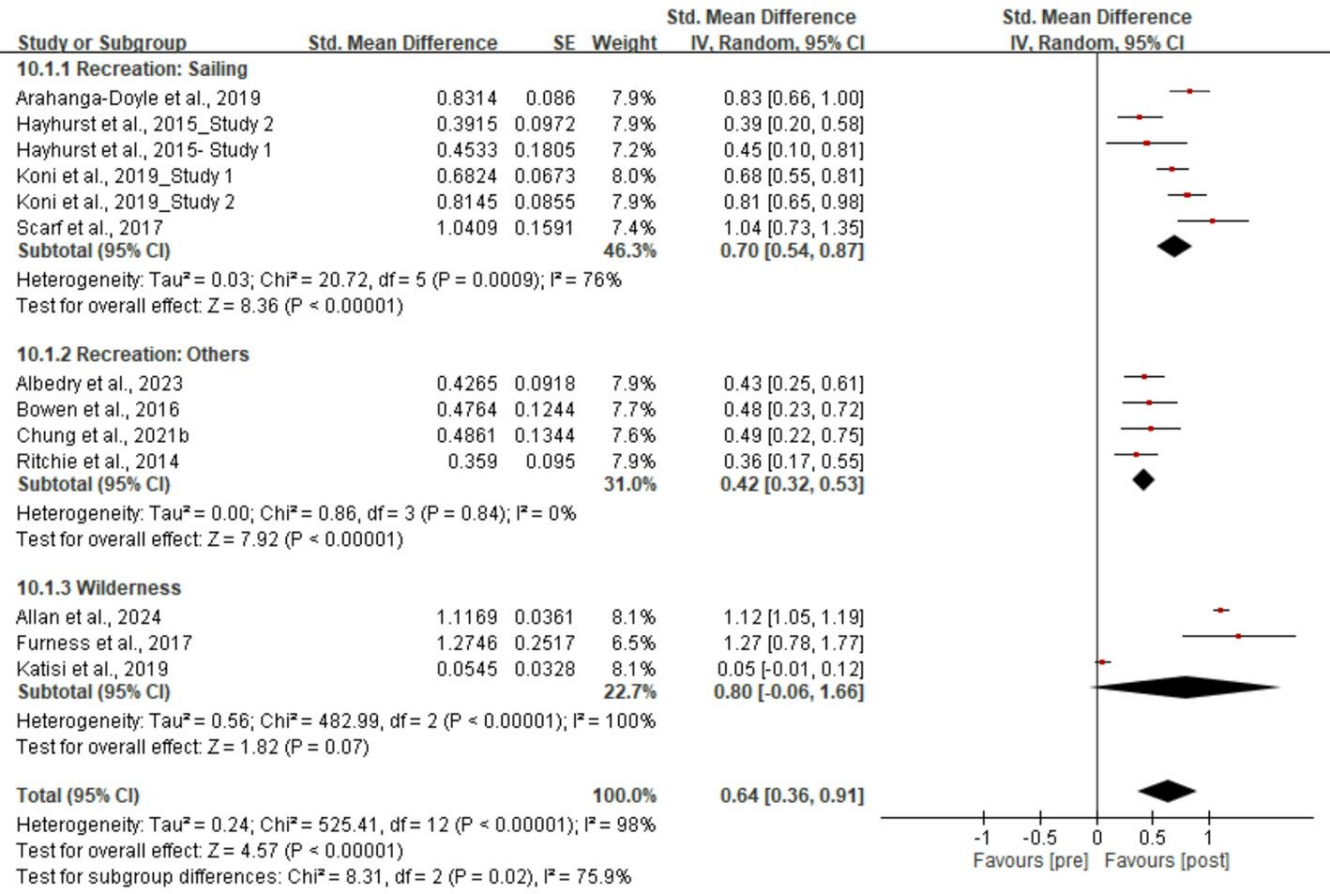
Pooled 95% CI SMD of NBIs on resilience

We summarised the outcomes and measurements of the 15 included studies (eTable 13 of Appendix). The primary meta-analysis included four controlled [40, 41, 43, 47] and nine single-group pre-post studies [35–37, 39, 43–46], while two single-group pre-post studies [38, 42] were excluded due to insufficient data. The pooled estimate (Fig. 2) revealed a significant moderate-to-large association between NBIs and resilience enhancement post-intervention (SMD = 0.64; 95% CI: 0.36 to 0.91; *p* < 0.001). Substantial heterogeneity was observed (I^2^ = 98%). On average, children who participated in NBIs scored 0.64 SDs higher on resilience measures compared to controls. Subgroup analysis of NBI content revealed that sailing [37,43,45.47] had a significant moderate association (SMD = 0.70; 95% CI: 0.54 to 0.87; *p* < 0.001; I^2^ = 76%), followed by non-specific bundle of recreational activities [35, 39, 40, 46] (SMD = 0.42; 95% CI: 0.32 to 0.53; *p* < 0.001; I^2^ = 0%), while the effect of wilderness experiences [36, 41, 44] was not significant.

### Sensitivity analyses

The first sensitivity analysis (eFigure 4 of Appendix) showed consistent pooled effect estimate using an autocorrelation of 0.5 (SMD = 0.64; 95% CI: 0.38 to 0.91; *p* < 0.001; I^2^ = 96%), supporting the robustness of the primary meta-analysis results by assuming a conservative autocorrelation of 0.75. The second sensitivity analysis, excluding single-group pre-post studies (eFigure 5 of Appendix), revealed a consistent significant moderate-to-large association (SMD = 0.79; 95% CI: 0.42 to 1.16; *p* < 0.001; I^2^ = 79%). The final sensitivity analysis, excluding studies with serious or critical risk of bias [35, 36, 39, 46] (eFigure 6 of Appendix), further reinforcing the robustness of the findings by demonstrating a consistent significant moderate-to-large association (SMD = 0.65; 95% CI: 0.36 to 0.94; *p* < 0.001; I^2^ = 96%).

### Supplementary analyses

To ensure the validity of the meta-analysis findings and address study design differences, supplementary analyses were conducted to separately analysed two distinct type of effect sizes. The first analysis (eFigure 5 of Appendix) assessed the between-group effect size (SMD = 0.79; 95% CI: 0.42 to 1.16; *p* < 0.001; I^2^ = 79%). The second analysis (eFigure 7 of Appendix) examined within-group pre–post effect sizes from intervention groups only in controlled trials and single-group pre-post studies. This revealed a consistent, significant, moderate-to-large association (SMD = 0.58; 95% CI: 0.34 to 0.83; *p* < 0.001; I^2^ = 98%).

### Narrative syntheses

Studies summarised narratively [38, 42], along with those providing quantitative evidence, all demonstrated enhanced resilience immediately post-intervention (Table 2). Four studies [36, 39, 40, 43] measured short-term follow-up effects (ranging from one to six months), while one study [46] assessed long-term follow-up effects (12 months). The follow-up assessments revealed inconclusive results and did not indicate a sustained resilience enhancement effect. Only one study indicated a significant small to moderate effect [40]. While sample sizes in this synthesis vary widely (19 to 650), several were relatively small, potentially limiting generalizability. Limited follow-up data also restricts the assessment of NBIs’ long-term effects.

**Table 2:** Summary of evidence of resilience across included studies

Study	Design	Instrument	Evidence of resilience		
			Meta-analysis	Within-group analysis	Narrative
Albedry et al. [35]	PPS	CD-RISC		✓	
Allan et al. [36]	PPS	CD-RISC		✓	
Arahanga-Doyle et al. [37]	PPS	RS		✓	
Boarini et al. [38]	PPS	BESSI-20			✓
Bowen et al. [39]	PPS	RQ		✓	
Chung et al. [40]	RCT	RS	✓	✓	
Furness et al. [41]	CT	RS	✓	✓	
Gillespie and Allen-Craig [42]	PPS	RS			✓
Hayhurst et al. [43]_Study 1	CT	RS	✓	✓	
Hayhurst et al. [43]_Study 2	PPS	RS		✓	
Katisi et al. [44]	PPS	CYRM-28		✓	
Koni et al. [45]_Study 1	PPS	RS		✓	
Koni et al. [45] _Study 2	PPS	RS			
Ritchie et al. [46]	PPS	RS		✓	
Scarf et al. [47]	CT	RS	✓	✓	

^1^RCT, randomised clinical trial; CS, controlled trial; PPS, pre-post study

^2^BESSI-20, the behavioural, emotional, and social skills inventory—observer report form; CD-RISC, the connor-davidson resilience scale; CYRM-28, the child and youth resilience measure; RQ, resilience questionnaire; RS, the Wagnild and Young’s resilience scale

## Discussion

This systematic review and meta-analysis found a significant association between NBIs and short-term improvements in children’s resilience, although results should be interpreted cautiously due to study design limitation. The importance of nature-based experiences for young children has long been recognised in philosophy by early educational theorists such as Froebel, Dewey, and Montessori, who emphasised the role of these experiences in young children’s health and development [48]. However, much of the existing research literature focuses on supporting children’s health rather than on the contributions of nature experiences to resilience [49]. Unlike earlier narrative reviews on NBIs and youth wellbeing [26, 27], our study synthesised quantitative findings specific to resilience in children under 18, marking a methodological and age-focused advancement in the field. This study is the first systematic review contributes early quantitative evidence suggesting an alternative community-based approach that may support the development of resilience by incorporating nature. Substantial heterogeneity was observed in our analyses, potentially attributable to variations in study designs, measuring instruments, NBI delivery methods, dosage, and the diverse social challenges faced by the participants. Nevertheless, meta-analyses were conducted as the directions of the included studies’ effects were generally consistent. Despite limitations such as limited follow-up data, a small number of studies, and reliance on one RCT alongside controlled trials and single-group pre-post studies, we employed the most robust methodology available and ensure transparency regarding the trade-offs between data inclusion and result validity to address gaps in the NBI literature.

All included studies incorporated adventure-based experiential learning experiences, which engage participants physically, emotionally, and mentally through designed activities [50, 51]. Adventure-based experiential learning fosters children’s resilience by providing opportunities to face challenges in a safe and supportive environment. High perceived risk and exposure to unusual settings intensify interpersonal and intrapersonal processes, further enhancing resilience [52]. Grounding adventure-based experiential learning in nature increases its effectiveness by providing greater autonomy than indoor spaces [53]. This autonomy allows children to take ownership of their actions, manage challenges, recover from setbacks, and foster personal transformation through positive reflection [54]. These experiences align with the Synergy Model of Resilience [55], which emphasises overcoming structured challenges to build “resistance to adversity” and achieve positive outcomes. A recent RCT aligns with this perspective, indicating that NBIs without adventure-based elements (e.g., mindfulness, art therapy, green-space walking) have minimal impact on resilience [56]. This suggests that adventure-based experiential learning, which combines physical challenge, nature immersion, and experiential learning, may play a unique role in supporting children’s development.

Our systematic review observed a notable overlap in terminology related to recreational activities within NBIs. It is essential to distinguish NBIs from recreational therapy, wilderness therapy, or adventure therapy. While these approaches also involve immersion in nature and the interplay between challenge and nature, they primarily focus on transformative experiences to build resilience [57–59]. In contrast, NBIs are grounded in intentional therapeutic processes specific to outdoor therapies [23]. Highlighting the nature-human kinship is crucial, as NBIs emphasise the therapeutic benefits of nature itself, rather than relying on outdoor recreation.

Regarding the core content used in NBI delivery, sailing showed some promise based on subgroup analysis on NBI content and its narratives emphasising nature-human kinship. Navigating while sailing requires understanding the wind and water within a social environment that demands teamwork, communication, and problem-solving skills [60]. These skills contribute significantly to developing social functioning [61], which is positively associated with resilience [62]. A recent systematic review highlights sailing benefits across physical, mental, and social domains in children and adolescents [63]. Notably, this review is the first to quantitatively suggest that sailing may help enhance resilience in children. However, this remains an early observation and not yet a proven conclusion. High-quality trials are needed to evaluate the effects of sailing on resilience beyond narrative-based findings [64].

### Implications for research

Recognising that NBIs provide developmental benefits associated with exposure to nature in young children [65], identifying a lack of studies focused on children aged 7–12 is a critical observation. This stage, crucial for developing social functioning skills as emphasised in Piaget’s theory [66], also represents an optimal period for fostering resilience [10, 67]. Future research should prioritise investigating the effects of NBIs on children aged 7–12, as this age group remains underrepresented in the existing literature. Targeting this developmental stage could provide valuable insights into age-specific impacts of NBIs on resilience across diverse life domains. While our review primarily focuses on resilience, it also notes its association with developmental outcomes such as self-esteem [37, 39, 40, 43], mental health outcomes such as depressive symptoms [39, 40], and broader intervention outcomes such as quality of life [38]. Future research should explore the relationship between resilience and these outcomes, considering resilience as a potential protective factor or contributor to promoting health among children. Our review provides valuable insights for children facing mental health and social challenges, as reflected in the included study participants (eTable 14 of Appendix). This aligns with existing literature, which suggests that resilience interventions are associated with improvements in the social integration of children with diverse needs [68].

Acknowledging the limitations of existing resilience research within the neurodiversity paradigm [69], the insights from this review may extend to children with diverse needs who face challenges similar to those discussed, such as autistic children [70] and children with cancer [71]. This aligns with literature suggesting that resilience is teachable and beneficial for individuals dealing with challenging circumstances [72]. This understanding paves the way for developing more effective NBIs that empower children to acquire the ability to bounce back from difficult but inevitable experiences through community-based initiatives.

### Limitations

This review highlights the evolving research on NBIs and children’s resilience, providing a foundation to address gaps, such as the long-term effect of NBIs and their impact on children outside the 13–17 age range. However, several limitations of this review should be acknowledged. First, only one RCT was included, with the remainder comprising non-randomised or single-group pre-post designs, limiting the strength of causal inferences. Notably, the predominance of single-group pre-post studies (11 out of 15) may introduce bias due to the absence of control groups, increasing susceptibility to confounding factors such as maturation, expectancy effects, or regression to the mean. As a result, the generalisability and strength of causal inferences drawn from the pooled estimates should be interpreted with caution. Second, a substantial number of included studies had a moderate to critical risk of bias (three studies rated as “critical”; see eFigure 3). Third, considerable heterogeneity was observed in the pooled analysis (I^2^ = 98%), which may reduce the precision of the effect estimate. Finally, follow-up data were limited across studies, making it difficult to assess the long-term sustainability of NBI effects on resilience.

Evidence suggesting that sailing enhances resilience remains preliminary. Future research should prioritise rigorous trials, expand participant demographics, explore long-term effects, and provide robust, generalisable evidence on the transformative potential of NBIs, such as sailing, for enhancing children’s resilience. While a comprehensive search strategy across multiple databases and grey literature was implemented, restricting the review to English-language publications may have introduced language bias. As cultural context significantly shapes perceptions of resilience [73], studies in non-western and diverse settings are crucial to broaden insights beyond predominantly western contexts.

## Conclusion

The review, the first meta-analysis of NBIs targeting children, provides preliminary evidence that NBIs involving group-based, adventure-based experiential learning may be associated with short-term improvements in children’s resilience. It represents early evidence suggesting a community-based approach that my support resilience development through nature. Sailing showed some promise in subgroup analysis and narrative synthesis emphasising nature-human kinship, though this remains an early observation, not a proven conclusion. High-quality trials are needed to evaluate the effects of sailing on resilience and address two key gaps: the long-term effect of NBIs and their impact on children outside the 13–17 age range.

## Supplementary Information

Below is the link to the electronic supplementary material.

Supplementary file1 (DOCX 992 kb)

## Data Availability

No datasets were generated or analysed during the current study.

## Abbreviations

CENTRAL: Cochrane central register of controlled trials
CI: Confidence interval
CICAHL: Cumulative index to nursing and allied health literature
ERIC: Education resources information center
NBI: Nature-based intervention
PICO: Population, intervention, control, and outcomes
PRISMA: Preferred reporting items for systematic reviews and meta-analyses
RCT: Randomised controlled trials
ROB: Risk of bias
RoB 2: The revised Cochrane risk-of-bias tool for randomized trials
ROBINS-I: The risk of bias in non-randomized studies–of interventions
SD: Standard deviation
SE: Standard error
SMD: Standardised mean difference
