# Supplementary material for: Nature-based interventions for enhancing resilience in children: a systematic review and meta-analysis
Source: Discov Ment Health. 2025 Jul 23;5(1):109. doi: 10.1007/s44192-025-00258-7 (PMC12287494; doi:10.1007/s44192-025-00258-7)

**Appendix Content**

eTable 1. Population, Intervention, Comparison, and Outcome (PICO) Table of Study Eligibility Criteria.

eTable 2. Study Search Strategy

eTable 3. CINAHL Search Results

eTable 4. CENTRAL Search Results

eTable 5. Embase Search Results

eTable 6. ERIC Search Results

eTable 7. Medline Search Results

eTable 8.APA PsycArticles Search Results

eTable 9. SPORTDiscus Search Results

eTable 10. Web of Science Search Results

eTable 11. Comprehensive Details of the GRADE Tool

eTable 12. Summary Description of the Characteristics of Included Studies

eTable 13. Summary Description of the Outcomes and Measurements of Included Studies

eFigure 1: Funnel Plot for Assessing Publication Bias

eFigure 2. Revised Cochrane Risk-of-Bias tool for randomized trials (RoB 2) Summary and Author Judgments of Low, Some concerns, and High Risk of Bias Across All Included RCTs studies (N = 1)

eFigure 3. Cochrane Risk of Bias in Non-randomized Studies – of Intervention (ROBINS-I) Summary and Author Judgments of Low, Moderate, serious, and Critical Risk of Bias Across All Included Non-RCTs Studies (N = 13)

eFigure 4. Sensitivity Analysis Using a Correlation of 0.5

eFigure 5. Sensitivity Analysis Excluding Single-Group Pre-Post Studies

eFigure 6. Sensitivity Analysis Excluding Studies with Serious or Critical Risk of Bias

eFigure 7. Pooled Effect Sizes for Within-Group Pre–Post Changes in Intervention Arms of Controlled Trials and Single-Group Pre–Post Studies

eTable 1. Population, intervention, Comparison, and Outcome (PICO) Table of Study Eligibility Criteria.


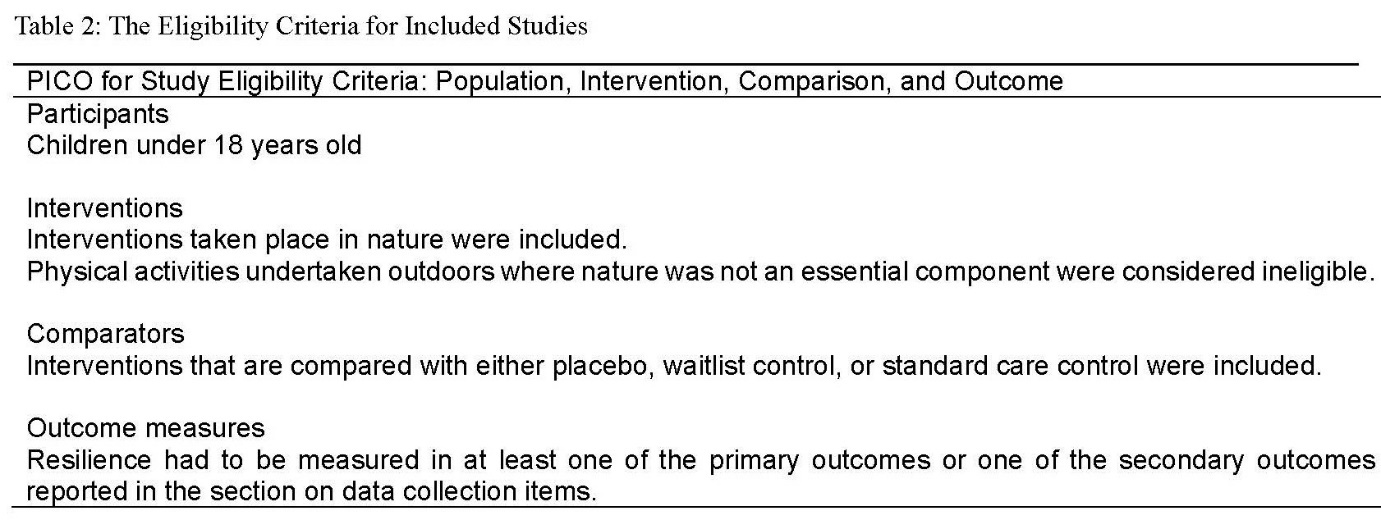


eTable 2. Study Search Strategy

| Search Concepts | MeSH | Keywords (add all possible synonyms, variations, and related terms) |
| --- | --- | --- |
| Resilience |  | Resilience OR psychological resilience OR grit |
| Nature-based |  | natur* OR nature-base* OR flow* OR adventure* OR expedition* OR experiential* OR outdoor* OR wilderness* OR walking OR running OR hiking OR swimming OR surf*OR water sports OR sailing OR kayak* OR rowing OR golf* |
| Intervention | Methods/ | Intervention* OR recreation* OR exercise*OR sport*OR education*OR physical* OR physical education and training OR therap* OR training* OR activit* OR experience* OR program* |

eTable 3. CINAHL Search Results

Tue, November 26, 2024 7:38:40 AM

| **#** | **Query** | **Limiters/Expanders** | **Last Run Via** | **Results** |
| --- | --- | --- | --- | --- |
| S62 | S58 AND S61 | Expanders - Apply equivalent subjects Search modes - Proximity | Interface - EBSCOhost Research Databases Search Screen - Advanced Search  Database - CINAHL Ultimate | 986 |
| S61 | S59 OR S60 | Expanders - Apply equivalent subjects Search modes - Proximity | Interface - EBSCOhost Research Databases Search Screen - Advanced Search  Database - CINAHL Ultimate | Display |
| S60 | "grit" | Expanders - Apply equivalent subjects Search modes - Proximity | Interface - EBSCOhost Research Databases Search Screen - Advanced Search  Database - CINAHL Ultimate | Display |
| S59 | "resilience" | Expanders - Apply equivalent subjects Search modes - Proximity | Interface - EBSCOhost Research Databases Search Screen - Advanced Search  Database - CINAHL Ultimate | Display |
| S58 | S42 AND S57 | Expanders - Apply equivalent subjects Search modes - Proximity | Interface - EBSCOhost Research Databases Search Screen - Advanced Search  Database - CINAHL Ultimate | Display |
| S57 | S43 OR S44 OR S45 OR S46 OR S47 OR S48 OR S49 OR S50 OR S51 OR S52 OR S53 OR S54 OR S55 OR S56 | Expanders - Apply equivalent subjects Search modes - Proximity | Interface - EBSCOhost Research Databases Search Screen - Advanced Search  Database - CINAHL Ultimate | Display |
| S56 | (MH "Golf") | Expanders - Apply equivalent subjects Search modes - Proximity | Interface - EBSCOhost Research Databases Search Screen - Advanced Search  Database - CINAHL Ultimate | Display |
| S55 | (MH "Rowing") | Expanders - Apply equivalent subjects Search modes - Proximity | Interface - EBSCOhost Research Databases Search Screen - Advanced Search  Database - CINAHL Ultimate | Display |
| S54 | "sailing" | Expanders - Apply equivalent subjects Search modes - Proximity | Interface - EBSCOhost Research Databases Search Screen - Advanced Search  Database - CINAHL Ultimate | Display |
| S53 | (MH "Aquatic Sports") | Expanders - Apply equivalent subjects Search modes - Proximity | Interface - EBSCOhost Research Databases Search Screen - Advanced Search  Database - CINAHL Ultimate | Display |
| S52 | (MH "Swimming") | Expanders - Apply equivalent subjects Search modes - Proximity | Interface - EBSCOhost Research Databases Search Screen - Advanced Search  Database - CINAHL Ultimate | Display |
| S51 | "hiking" | Expanders - Apply equivalent subjects Search modes - Proximity | Interface - EBSCOhost Research Databases Search Screen - Advanced Search  Database - CINAHL Ultimate | Display |
| S50 | (MH "Running") | Expanders - Apply equivalent subjects Search modes - Proximity | Interface - EBSCOhost Research Databases Search Screen - Advanced Search  Database - CINAHL Ultimate | Display |
| S49 | (MH "Walking") | Expanders - Apply equivalent subjects Search modes - Proximity | Interface - EBSCOhost Research Databases Search Screen - Advanced Search  Database - CINAHL Ultimate | Display |
| S48 | (MH "Wilderness Experience") OR “outdoor” | Expanders - Apply equivalent subjects Search modes - Proximity | Interface - EBSCOhost Research Databases Search Screen - Advanced Search  Database - CINAHL Ultimate | Display |
| S47 | (MH "Experiential Learning") OR "expedition" | Expanders - Apply equivalent subjects Search modes - Proximity | Interface - EBSCOhost Research Databases Search Screen - Advanced Search  Database - CINAHL Ultimate | Display |
| S46 | (MH "Wilderness Experience") OR (MH "Sports") | Expanders - Apply equivalent subjects Search modes - Proximity | Interface - EBSCOhost Research Databases Search Screen - Advanced Search  Database - CINAHL Ultimate | Display |
| S45 | "flow*" | Expanders - Apply equivalent subjects Search modes - Proximity | Interface - EBSCOhost Research Databases Search Screen - Advanced Search  Database - CINAHL Ultimate | Display |
| S44 | "nature-based" | Expanders - Apply equivalent subjects Search modes - Proximity | Interface - EBSCOhost Research Databases Search Screen - Advanced Search  Database - CINAHL Ultimate | Display |
| S43 | "natur*" | Expanders - Apply equivalent subjects Search modes - Proximity | Interface - EBSCOhost Research Databases Search Screen - Advanced Search  Database - CINAHL Ultimate | Display |
| S42 | S32 OR S33 OR S34 OR S35 OR S36 OR S37 OR S38 OR S39 OR S40 OR S41 | Expanders - Apply equivalent subjects Search modes - Proximity | Interface - EBSCOhost Research Databases Search Screen - Advanced Search  Database - CINAHL Ultimate | Display |
| S41 | "program*" | Expanders - Apply equivalent subjects Search modes - Proximity | Interface - EBSCOhost Research Databases Search Screen - Advanced Search  Database - CINAHL Ultimate | Display |
| S40 | "experience*" | Expanders - Apply equivalent subjects Search modes - Proximity | Interface - EBSCOhost Research Databases Search Screen - Advanced Search  Database - CINAHL Ultimate | Display |
| S39 | "activit*" | Expanders - Apply equivalent subjects Search modes - Proximity | Interface - EBSCOhost Research Databases Search Screen - Advanced Search  Database - CINAHL Ultimate | Display |
| S38 | "training" | Expanders - Apply equivalent subjects Search modes - Proximity | Interface - EBSCOhost Research Databases Search Screen - Advanced Search  Database - CINAHL Ultimate | Display |
| S37 | (MH "Physical Activity") | Expanders - Apply equivalent subjects Search modes - Proximity | Interface - EBSCOhost Research Databases Search Screen - Advanced Search  Database - CINAHL Ultimate | Display |
| S36 | (MH "Education") | Expanders - Apply equivalent subjects Search modes - Proximity | Interface - EBSCOhost Research Databases Search Screen - Advanced Search  Database - CINAHL Ultimate | Display |
| S35 | "sport" | Expanders - Apply equivalent subjects Search modes - Proximity | Interface - EBSCOhost Research Databases Search Screen - Advanced Search  Database - CINAHL Ultimate | Display |
| S34 | (MH "Exercise") | Expanders - Apply equivalent subjects Search modes - Proximity | Interface - EBSCOhost Research Databases Search Screen - Advanced Search  Database - CINAHL Ultimate | Display |
| S33 | (MH "Recreational Therapy") OR (MH "Recreation") | Expanders - Apply equivalent subjects Search modes - Proximity | Interface - EBSCOhost Research Databases Search Screen - Advanced Search  Database - CINAHL Ultimate | Display |
| S32 | (MH "Intervention Trials") OR (MH "Experimental Studies") | Expanders - Apply equivalent subjects Search modes - Proximity | Interface - EBSCOhost Research Databases Search Screen - Advanced Search  Database - CINAHL Ultimate | Display |
| S31 | S27 AND S30 | Expanders - Apply equivalent subjects Search modes - Proximity | Interface - EBSCOhost Research Databases Search Screen - Advanced Search  Database - CINAHL Ultimate | 986 |
| S30 | S28 OR S29 | Expanders - Apply equivalent subjects Search modes - Proximity | Interface - EBSCOhost Research Databases Search Screen - Advanced Search  Database - CINAHL Ultimate | 24,337 |
| S29 | "grit" | Expanders - Apply equivalent subjects Search modes - Proximity | Interface - EBSCOhost Research Databases Search Screen - Advanced Search  Database - CINAHL Ultimate | 739 |
| S28 | "resilience" | Expanders - Apply equivalent subjects Search modes - Proximity | Interface - EBSCOhost Research Databases Search Screen - Advanced Search  Database - CINAHL Ultimate | 23,742 |
| S27 | S11 AND S26 | Expanders - Apply equivalent subjects Search modes - Proximity | Interface - EBSCOhost Research Databases Search Screen - Advanced Search  Database - CINAHL Ultimate | 119,758 |
| S26 | S12 OR S13 OR S14 OR S15 OR S16 OR S17 OR S18 OR S19 OR S20 OR S21 OR S22 OR S23 OR S24 OR S25 | Expanders - Apply equivalent subjects Search modes - Proximity | Interface - EBSCOhost Research Databases Search Screen - Advanced Search  Database - CINAHL Ultimate | 355,436 |
| S25 | (MH "Golf") | Expanders - Apply equivalent subjects Search modes - Proximity | Interface - EBSCOhost Research Databases Search Screen - Advanced Search  Database - CINAHL Ultimate | 1,395 |
| S24 | (MH "Rowing") | Expanders - Apply equivalent subjects Search modes - Proximity | Interface - EBSCOhost Research Databases Search Screen - Advanced Search  Database - CINAHL Ultimate | 1,114 |
| S23 | "sailing" | Expanders - Apply equivalent subjects Search modes - Proximity | Interface - EBSCOhost Research Databases Search Screen - Advanced Search  Database - CINAHL Ultimate | 1,064 |
| S22 | (MH "Aquatic Sports") | Expanders - Apply equivalent subjects Search modes - Proximity | Interface - EBSCOhost Research Databases Search Screen - Advanced Search  Database - CINAHL Ultimate | 1,081 |
| S21 | (MH "Swimming") | Expanders - Apply equivalent subjects Search modes - Proximity | Interface - EBSCOhost Research Databases Search Screen - Advanced Search  Database - CINAHL Ultimate | 5,031 |
| S20 | "hiking" | Expanders - Apply equivalent subjects Search modes - Proximity | Interface - EBSCOhost Research Databases Search Screen - Advanced Search  Database - CINAHL Ultimate | 333 |
| S19 | (MH "Running") | Expanders - Apply equivalent subjects Search modes - Proximity | Interface - EBSCOhost Research Databases Search Screen - Advanced Search  Database - CINAHL Ultimate | 10,892 |
| S18 | (MH "Walking") | Expanders - Apply equivalent subjects Search modes - Proximity | Interface - EBSCOhost Research Databases Search Screen - Advanced Search  Database - CINAHL Ultimate | 26,041 |
| S17 | (MH "Wilderness Experience") OR "outdoor" | Expanders - Apply equivalent subjects Search modes - Proximity | Interface - EBSCOhost Research Databases Search Screen - Advanced Search  Database - CINAHL Ultimate | 6,063 |
| S16 | (MH "Experiential Learning") OR "expedition" | Expanders - Apply equivalent subjects Search modes - Proximity | Interface - EBSCOhost Research Databases Search Screen - Advanced Search  Database - CINAHL Ultimate | 4,289 |
| S15 | (MH "Wilderness Experience") OR (MH "Sports") | Expanders - Apply equivalent subjects Search modes - Proximity | Interface - EBSCOhost Research Databases Search Screen - Advanced Search  Database - CINAHL Ultimate | 10,494 |
| S14 | "flow*" | Expanders - Apply equivalent subjects Search modes - Proximity | Interface - EBSCOhost Research Databases Search Screen - Advanced Search  Database - CINAHL Ultimate | 107,240 |
| S13 | "nature-based" | Expanders - Apply equivalent subjects Search modes - Proximity | Interface - EBSCOhost Research Databases Search Screen - Advanced Search  Database - CINAHL Ultimate | 298 |
| S12 | "natur*" | Expanders - Apply equivalent subjects Search modes - Proximity | Interface - EBSCOhost Research Databases Search Screen - Advanced Search  Database - CINAHL Ultimate | 191,630 |
| S11 | S1 OR S2 OR S3 OR S4 OR S5 OR S6 OR S7 OR S8 OR S9 OR S10 | Expanders - Apply equivalent subjects Search modes - Proximity | Interface - EBSCOhost Research Databases Search Screen - Advanced Search  Database - CINAHL Ultimate | 1,725,300 |
| S10 | "program*" | Expanders - Apply equivalent subjects Search modes - Proximity | Interface - EBSCOhost Research Databases Search Screen - Advanced Search  Database - CINAHL Ultimate | 613,374 |
| S9 | "experience*" | Expanders - Apply equivalent subjects Search modes - Proximity | Interface - EBSCOhost Research Databases Search Screen - Advanced Search  Database - CINAHL Ultimate | 584,591 |
| S8 | "activit*" | Expanders - Apply equivalent subjects Search modes - Proximity | Interface - EBSCOhost Research Databases Search Screen - Advanced Search  Database - CINAHL Ultimate | 482,021 |
| S7 | "training" | Expanders - Apply equivalent subjects Search modes - Proximity | Interface - EBSCOhost Research Databases Search Screen - Advanced Search  Database - CINAHL Ultimate | 286,385 |
| S6 | (MH "Physical Activity") | Expanders - Apply equivalent subjects Search modes - Proximity | Interface - EBSCOhost Research Databases Search Screen - Advanced Search  Database - CINAHL Ultimate | 56,211 |
| S5 | (MH "Education") | Expanders - Apply equivalent subjects Search modes - Proximity | Interface - EBSCOhost Research Databases Search Screen - Advanced Search  Database - CINAHL Ultimate | 11,552 |
| S4 | "sport" | Expanders - Apply equivalent subjects Search modes - Proximity | Interface - EBSCOhost Research Databases Search Screen - Advanced Search  Database - CINAHL Ultimate | 29,363 |
| S3 | (MH "Exercise") | Expanders - Apply equivalent subjects Search modes - Proximity | Interface - EBSCOhost Research Databases Search Screen - Advanced Search  Database - CINAHL Ultimate | 61,834 |
| S2 | (MH "Recreational Therapy") OR (MH "Recreation") | Expanders - Apply equivalent subjects Search modes - Proximity | Interface - EBSCOhost Research Databases Search Screen - Advanced Search  Database - CINAHL Ultimate | 6,932 |
| S1 | (MH "Intervention Trials") OR (MH "Experimental Studies") | Expanders - Apply equivalent subjects Search modes - Proximity | Interface - EBSCOhost Research Databases Search Screen - Advanced Search  Database - CINAHL Ultimate | 37,780 |

eTable 4. CENTRAL Search Results

Date Run: 25/11/2024 18:17:13

| ID | Search Hits |  |
| --- | --- | --- |
| #1 | intervention* | 664246 |
| #2 | recreation* | 6020 |
| #3 | exercise* | 158272 |
| #4 | sport | 15843 |
| #5 | education* | 134602 |
| #6 | physical* | 196035 |
| #7 | physical education and training | 10513 |
| #8 | therap* | 1033083 |
| #9 | activit* | 240563 |
| #10 | experience* | 152063 |
| #11 | program* | 189704 |
| #12 | #1 OR #2 OR #3 OR #4 OR #5 OR #6 OR #7 OR #8 OR #9 OR #10 OR #11 | 1598389 |
| #13 | natur* | 56106 |
| #14 | nature-based | 174 |
| #15 | flow* | 77139 |
| #16 | adventure* | 234 |
| #17 | Expedition* | 176 |
| #18 | experiential* | 1667 |
| #19 | outdoor* | 2254 |
| #20 | wilderness* | 139 |
| #21 | walking* | 33500 |
| #22 | running* | 10400 |
| #23 | hiking* | 135 |
| #24 | swimming* | 1672 |
| #25 | surfing* | 62 |
| #26 | water sport | 848 |
| #27 | sailing* | 73 |
| #28 | sail* | 802 |
| #29 | rowing* | 537 |
| #30 | golf* | 481 |
| #31 | #13 OR #14 OR #15 OR #16 OR #17 OR #18 OR #19 OR #20 OR #21 OR #22 OR #23 OR #24 OR #25 OR #26 OR #27 OR #28 OR #29 OR #30 | 170349 |
| #32 | #12 AND #31 | 141296 |
| #33 | MeSH descriptor: [Resilience, Psychological] explode all trees | 503 |
| #34 | #32 AND #33 | 31 |

eTable 5. Embase Search Results3

EMBASE via Ovid, Embase 1910 to November 2024

| 1 | Resilience, Psychological/ | 12821 |
| --- | --- | --- |
| 2 | grit.mp. | 3481 |
| 3 | 1 or 2 | 16223 |
| 4 | natur*.mp. | 1878254 |
| 5 | nature-base*.mp. | 2303 |
| 6 | flow*.mp. | 1810585 |
| 7 | adventure*.mp. | 2980 |
| 8 | Expeditions/ | 67840 |
| 9 | outdoor*.mp. | 44598 |
| 10 | Wilderness/ | 614 |
| 11 | Walking/ | 93193 |
| 12 | Running/ | 43460 |
| 13 | hiking.mp. | 1424 |
| 14 | Swimming/ | 29496 |
| 15 | surf*.mp. | 1907294 |
| 16 | Water Sports/ | 427 |
| 17 | sailing.mp. | 1207 |
| 18 | kayak*.mp. | 4367 |
| 19 | rowing.mp. | 2801 |
| 20 | Golf/ | 1350 |
| 21 | 4 or 5 or 6 or 7 or 8 or 9 or 10 or 11 or 12 or 13 or 14 or 15 or 16 or 17 or 18 or 19 or 20 | 5414742 |
| 22 | 3 and 21 | 2858 |
| 23 | Intervention*.mp. | 2199228 |
| 24 | Recreation/ | 21748 |
| 25 | Exercise/ | 377473 |
| 26 | Education/ | 508753 |
| 27 | "Physical Education and Training"/ | 13228 |
| 28 | experience*.mp. | 2148381 |
| 29 | program*.mp. | 1936348 |
| 30 | activit*.mp. | 5788343 |
| 31 | 23 or 24 or 25 or 26 or 27 or 28 or 29 or 30 | 11119145 |
| 32 | 22 and 31 | 753 |

eTable 6. ERIC Search Results

Tue, November 26, 2024 7:46:34 AM

| **#** | **Query** | **Limiters/Expanders** | **Last Run Via** | **Results** |
| --- | --- | --- | --- | --- |
| S62 | S58 AND S61 | Expanders - Apply equivalent subjects Search modes - Proximity | Interface - EBSCOhost Research Databases Search Screen - Advanced Search  Database - CINAHL Ultimate | 986 |
| S61 | S59 OR S60 | Expanders - Apply equivalent subjects Search modes - Proximity | Interface - EBSCOhost Research Databases Search Screen - Advanced Search  Database - CINAHL Ultimate | Display |
| S60 | "grit" | Expanders - Apply equivalent subjects Search modes - Proximity | Interface - EBSCOhost Research Databases Search Screen - Advanced Search  Database - CINAHL Ultimate | Display |
| S59 | "resilience" | Expanders - Apply equivalent subjects Search modes - Proximity | Interface - EBSCOhost Research Databases Search Screen - Advanced Search  Database - CINAHL Ultimate | Display |
| S58 | S42 AND S57 | Expanders - Apply equivalent subjects Search modes - Proximity | Interface - EBSCOhost Research Databases Search Screen - Advanced Search  Database - CINAHL Ultimate | Display |
| S57 | S43 OR S44 OR S45 OR S46 OR S47 OR S48 OR S49 OR S50 OR S51 OR S52 OR S53 OR S54 OR S55 OR S56 | Expanders - Apply equivalent subjects Search modes - Proximity | Interface - EBSCOhost Research Databases Search Screen - Advanced Search  Database - CINAHL Ultimate | Display |
| S56 | (MH "Golf") | Expanders - Apply equivalent subjects Search modes - Proximity | Interface - EBSCOhost Research Databases Search Screen - Advanced Search  Database - CINAHL Ultimate | Display |
| S55 | (MH "Rowing") | Expanders - Apply equivalent subjects Search modes - Proximity | Interface - EBSCOhost Research Databases Search Screen - Advanced Search  Database - CINAHL Ultimate | Display |
| S54 | "sailing" | Expanders - Apply equivalent subjects Search modes - Proximity | Interface - EBSCOhost Research Databases Search Screen - Advanced Search  Database - CINAHL Ultimate | Display |
| S53 | (MH "Aquatic Sports") | Expanders - Apply equivalent subjects Search modes - Proximity | Interface - EBSCOhost Research Databases Search Screen - Advanced Search  Database - CINAHL Ultimate | Display |
| S52 | (MH "Swimming") | Expanders - Apply equivalent subjects Search modes - Proximity | Interface - EBSCOhost Research Databases Search Screen - Advanced Search  Database - CINAHL Ultimate | Display |
| S51 | "hiking" | Expanders - Apply equivalent subjects Search modes - Proximity | Interface - EBSCOhost Research Databases Search Screen - Advanced Search  Database - CINAHL Ultimate | Display |
| S50 | (MH "Running") | Expanders - Apply equivalent subjects Search modes - Proximity | Interface - EBSCOhost Research Databases Search Screen - Advanced Search  Database - CINAHL Ultimate | Display |
| S49 | (MH "Walking") | Expanders - Apply equivalent subjects Search modes - Proximity | Interface - EBSCOhost Research Databases Search Screen - Advanced Search  Database - CINAHL Ultimate | Display |
| S48 | (MH "Wilderness Experience") OR “outdoor” | Expanders - Apply equivalent subjects Search modes - Proximity | Interface - EBSCOhost Research Databases Search Screen - Advanced Search  Database - CINAHL Ultimate | Display |
| S47 | (MH "Experiential Learning") OR "expedition" | Expanders - Apply equivalent subjects Search modes - Proximity | Interface - EBSCOhost Research Databases Search Screen - Advanced Search  Database - CINAHL Ultimate | Display |
| S46 | (MH "Wilderness Experience") OR (MH "Sports") | Expanders - Apply equivalent subjects Search modes - Proximity | Interface - EBSCOhost Research Databases Search Screen - Advanced Search  Database - CINAHL Ultimate | Display |
| S45 | "flow*" | Expanders - Apply equivalent subjects Search modes - Proximity | Interface - EBSCOhost Research Databases Search Screen - Advanced Search  Database - CINAHL Ultimate | Display |
| S44 | "nature-based" | Expanders - Apply equivalent subjects Search modes - Proximity | Interface - EBSCOhost Research Databases Search Screen - Advanced Search  Database - CINAHL Ultimate | Display |
| S43 | "natur*" | Expanders - Apply equivalent subjects Search modes - Proximity | Interface - EBSCOhost Research Databases Search Screen - Advanced Search  Database - CINAHL Ultimate | Display |
| S42 | S32 OR S33 OR S34 OR S35 OR S36 OR S37 OR S38 OR S39 OR S40 OR S41 | Expanders - Apply equivalent subjects Search modes - Proximity | Interface - EBSCOhost Research Databases Search Screen - Advanced Search  Database - CINAHL Ultimate | Display |
| S41 | "program*" | Expanders - Apply equivalent subjects Search modes - Proximity | Interface - EBSCOhost Research Databases Search Screen - Advanced Search  Database - CINAHL Ultimate | Display |
| S40 | "experience*" | Expanders - Apply equivalent subjects Search modes - Proximity | Interface - EBSCOhost Research Databases Search Screen - Advanced Search  Database - CINAHL Ultimate | Display |
| S39 | "activit*" | Expanders - Apply equivalent subjects Search modes - Proximity | Interface - EBSCOhost Research Databases Search Screen - Advanced Search  Database - CINAHL Ultimate | Display |
| S38 | "training" | Expanders - Apply equivalent subjects Search modes - Proximity | Interface - EBSCOhost Research Databases Search Screen - Advanced Search  Database - CINAHL Ultimate | Display |
| S37 | (MH "Physical Activity") | Expanders - Apply equivalent subjects Search modes - Proximity | Interface - EBSCOhost Research Databases Search Screen - Advanced Search  Database - CINAHL Ultimate | Display |
| S36 | (MH "Education") | Expanders - Apply equivalent subjects Search modes - Proximity | Interface - EBSCOhost Research Databases Search Screen - Advanced Search  Database - CINAHL Ultimate | Display |
| S35 | "sport" | Expanders - Apply equivalent subjects Search modes - Proximity | Interface - EBSCOhost Research Databases Search Screen - Advanced Search  Database - CINAHL Ultimate | Display |
| S34 | (MH "Exercise") | Expanders - Apply equivalent subjects Search modes - Proximity | Interface - EBSCOhost Research Databases Search Screen - Advanced Search  Database - CINAHL Ultimate | Display |
| S33 | (MH "Recreational Therapy") OR (MH "Recreation") | Expanders - Apply equivalent subjects Search modes - Proximity | Interface - EBSCOhost Research Databases Search Screen - Advanced Search  Database - CINAHL Ultimate | Display |
| S32 | (MH "Intervention Trials") OR (MH "Experimental Studies") | Expanders - Apply equivalent subjects Search modes - Proximity | Interface - EBSCOhost Research Databases Search Screen - Advanced Search  Database - CINAHL Ultimate | Display |
| S31 | S27 AND S30 | Expanders - Apply equivalent subjects Search modes - Proximity | Interface - EBSCOhost Research Databases Search Screen - Advanced Search  Database - CINAHL Ultimate | 986 |
| S30 | S28 OR S29 | Expanders - Apply equivalent subjects Search modes - Proximity | Interface - EBSCOhost Research Databases Search Screen - Advanced Search  Database - CINAHL Ultimate | 24,337 |
| S29 | "grit" | Expanders - Apply equivalent subjects Search modes - Proximity | Interface - EBSCOhost Research Databases Search Screen - Advanced Search  Database - CINAHL Ultimate | 739 |
| S28 | "resilience" | Expanders - Apply equivalent subjects Search modes - Proximity | Interface - EBSCOhost Research Databases Search Screen - Advanced Search  Database - CINAHL Ultimate | 23,742 |
| S27 | S11 AND S26 | Expanders - Apply equivalent subjects Search modes - Proximity | Interface - EBSCOhost Research Databases Search Screen - Advanced Search  Database - CINAHL Ultimate | 119,758 |
| S26 | S12 OR S13 OR S14 OR S15 OR S16 OR S17 OR S18 OR S19 OR S20 OR S21 OR S22 OR S23 OR S24 OR S25 | Expanders - Apply equivalent subjects Search modes - Proximity | Interface - EBSCOhost Research Databases Search Screen - Advanced Search  Database - CINAHL Ultimate | 355,436 |
| S25 | (MH "Golf") | Expanders - Apply equivalent subjects Search modes - Proximity | Interface - EBSCOhost Research Databases Search Screen - Advanced Search  Database - CINAHL Ultimate | 1,395 |
| S24 | (MH "Rowing") | Expanders - Apply equivalent subjects Search modes - Proximity | Interface - EBSCOhost Research Databases Search Screen - Advanced Search  Database - CINAHL Ultimate | 1,114 |
| S23 | "sailing" | Expanders - Apply equivalent subjects Search modes - Proximity | Interface - EBSCOhost Research Databases Search Screen - Advanced Search  Database - CINAHL Ultimate | 1,064 |
| S22 | (MH "Aquatic Sports") | Expanders - Apply equivalent subjects Search modes - Proximity | Interface - EBSCOhost Research Databases Search Screen - Advanced Search  Database - CINAHL Ultimate | 1,081 |
| S21 | (MH "Swimming") | Expanders - Apply equivalent subjects Search modes - Proximity | Interface - EBSCOhost Research Databases Search Screen - Advanced Search  Database - CINAHL Ultimate | 5,031 |
| S20 | "hiking" | Expanders - Apply equivalent subjects Search modes - Proximity | Interface - EBSCOhost Research Databases Search Screen - Advanced Search  Database - CINAHL Ultimate | 333 |
| S19 | (MH "Running") | Expanders - Apply equivalent subjects Search modes - Proximity | Interface - EBSCOhost Research Databases Search Screen - Advanced Search  Database - CINAHL Ultimate | 10,892 |
| S18 | (MH "Walking") | Expanders - Apply equivalent subjects Search modes - Proximity | Interface - EBSCOhost Research Databases Search Screen - Advanced Search  Database - CINAHL Ultimate | 26,041 |
| S17 | (MH "Wilderness Experience") OR "outdoor" | Expanders - Apply equivalent subjects Search modes - Proximity | Interface - EBSCOhost Research Databases Search Screen - Advanced Search  Database - CINAHL Ultimate | 6,063 |
| S16 | (MH "Experiential Learning") OR "expedition" | Expanders - Apply equivalent subjects Search modes - Proximity | Interface - EBSCOhost Research Databases Search Screen - Advanced Search  Database - CINAHL Ultimate | 4,289 |
| S15 | (MH "Wilderness Experience") OR (MH "Sports") | Expanders - Apply equivalent subjects Search modes - Proximity | Interface - EBSCOhost Research Databases Search Screen - Advanced Search  Database - CINAHL Ultimate | 10,494 |
| S14 | "flow*" | Expanders - Apply equivalent subjects Search modes - Proximity | Interface - EBSCOhost Research Databases Search Screen - Advanced Search  Database - CINAHL Ultimate | 107,240 |
| S13 | "nature-based" | Expanders - Apply equivalent subjects Search modes - Proximity | Interface - EBSCOhost Research Databases Search Screen - Advanced Search  Database - CINAHL Ultimate | 298 |
| S12 | "natur*" | Expanders - Apply equivalent subjects Search modes - Proximity | Interface - EBSCOhost Research Databases Search Screen - Advanced Search  Database - CINAHL Ultimate | 191,630 |
| S11 | S1 OR S2 OR S3 OR S4 OR S5 OR S6 OR S7 OR S8 OR S9 OR S10 | Expanders - Apply equivalent subjects Search modes - Proximity | Interface - EBSCOhost Research Databases Search Screen - Advanced Search  Database - CINAHL Ultimate | 1,725,300 |
| S10 | "program*" | Expanders - Apply equivalent subjects Search modes - Proximity | Interface - EBSCOhost Research Databases Search Screen - Advanced Search  Database - CINAHL Ultimate | 613,374 |
| S9 | "experience*" | Expanders - Apply equivalent subjects Search modes - Proximity | Interface - EBSCOhost Research Databases Search Screen - Advanced Search  Database - CINAHL Ultimate | 584,591 |
| S8 | "activit*" | Expanders - Apply equivalent subjects Search modes - Proximity | Interface - EBSCOhost Research Databases Search Screen - Advanced Search  Database - CINAHL Ultimate | 482,021 |
| S7 | "training" | Expanders - Apply equivalent subjects Search modes - Proximity | Interface - EBSCOhost Research Databases Search Screen - Advanced Search  Database - CINAHL Ultimate | 286,385 |
| S6 | (MH "Physical Activity") | Expanders - Apply equivalent subjects Search modes - Proximity | Interface - EBSCOhost Research Databases Search Screen - Advanced Search  Database - CINAHL Ultimate | 56,211 |
| S5 | (MH "Education") | Expanders - Apply equivalent subjects Search modes - Proximity | Interface - EBSCOhost Research Databases Search Screen - Advanced Search  Database - CINAHL Ultimate | 11,552 |
| S4 | "sport" | Expanders - Apply equivalent subjects Search modes - Proximity | Interface - EBSCOhost Research Databases Search Screen - Advanced Search  Database - CINAHL Ultimate | 29,363 |
| S3 | (MH "Exercise") | Expanders - Apply equivalent subjects Search modes - Proximity | Interface - EBSCOhost Research Databases Search Screen - Advanced Search  Database - CINAHL Ultimate | 61,834 |
| S2 | (MH "Recreational Therapy") OR (MH "Recreation") | Expanders - Apply equivalent subjects Search modes - Proximity | Interface - EBSCOhost Research Databases Search Screen - Advanced Search  Database - CINAHL Ultimate | 6,932 |
| S1 | (MH "Intervention Trials") OR (MH "Experimental Studies") | Expanders - Apply equivalent subjects Search modes - Proximity | Interface - EBSCOhost Research Databases Search Screen - Advanced Search  Database - CINAHL Ultimate | 37,780 |

eTable 7. Medline Search Results

Medline via Ovid, Ovid MEDLINE(R) <1946 to November 2024>

| 1 | Resilience, Psychological/ | 10758 |
| --- | --- | --- |
| 2 | grit.mp. | 2666 |
| 3 | 1 or 2 | 13357 |
| 4 | natur*.mp. | 1230865 |
| 5 | nature-base*.mp. | 1553 |
| 6 | flow*.mp. | 942247 |
| 7 | adventure*.mp. | 2086 |
| 8 | Expeditions/ | 1761 |
| 9 | outdoor*.mp. | 28796 |
| 10 | Wilderness/ | 369 |
| 11 | Walking/ | 44406 |
| 12 | Running/ | 24359 |
| 13 | hiking.mp. | 812 |
| 14 | Swimming/ | 20723 |
| 15 | surf*.mp. | 1275312 |
| 16 | Water Sports/ | 657 |
| 17 | sailing.mp. | 736 |
| 18 | kayak*.mp. | 466 |
| 19 | rowing.mp. | 1507 |
| 20 | Golf/ | 1378 |
| 21 | 4 or 5 or 6 or 7 or 8 or 9 or 10 or 11 or 12 or 13 or 14 or 15 or 16 or 17 or 18 or 19 or 20 | 3325526 |
| 22 | 3 and 21 | 2416 |
| 23 | Intervention*.mp. | 1310386 |
| 24 | Recreation/ | 7476 |
| 25 | Exercise/ | 154194 |
| 26 | Education/ | 21630 |
| 27 | "Physical Education and Training"/ | 14387 |
| 28 | experience*.mp. | 1265095 |
| 29 | program*.mp. | 1131901 |
| 30 | activit*.mp. | 3562188 |
| 31 | 23 or 24 or 25 or 26 or 27 or 28 or 29 or 30 | 6579656 |
| 32 | 22 and 31 | 588 |

eTable 8.APA PsycArticles Search Results

APA PsycArticles via Ovid

| 1 | Resilience, Psychological/ | 398 |
| --- | --- | --- |
| 2 | grit.mp. | 747 |
| 3 | 1 or 2 | 1131 |
| 4 | natur*.mp. | 108653 |
| 5 | nature-base*.mp. | 291 |
| 6 | flow*.mp. | 18598 |
| 7 | adventure*.mp. | 1362 |
| 8 | Expeditions/ | 95 |
| 9 | outdoor*.mp. | 2213 |
| 10 | Wilderness/ | 248 |
| 11 | Walking/ | 6102 |
| 12 | Running/ | 14531 |
| 13 | hiking.mp. | 293 |
| 14 | Swimming/ | 1794 |
| 15 | surf*.mp. | 15871 |
| 16 | Water Sports/ | 0 |
| 17 | sailing.mp. | 218 |
| 18 | kayak*.mp. | 64 |
| 19 | rowing.mp. | 138 |
| 20 | Golf/ | 753 |
| 21 | 4 or 5 or 6 or 7 or 8 or 9 or 10 or 11 or 12 or 13 or 14 or 15 or 16 or 17 or 18 or 19 or 20 | 116994 |
| 22 | 3 and 21 | 604 |
| 23 | Intervention*.mp. | 62312 |
| 24 | Recreation/ | 1979 |
| 25 | Exercise/ | 17071 |
| 26 | Education/ | 68096 |
| 27 | "Physical Education and Training"/ | 0 |
| 28 | experience*.mp. | 120864 |
| 29 | program*.mp. | 82593 |
| 30 | activit*.mp. | 78210 |
| 31 | 23 or 24 or 25 or 26 or 27 or 28 or 29 or 30 | 157149 |
| 32 | 22 and 31 | 559 |

eTable 9. SPORTDiscus Search Results

Tue, November 26, 2024 7:49:51 AM

| **#** | **Query** | **Limiters/Expanders** | **Last Run Via** | **Results** |
| --- | --- | --- | --- | --- |
| S62 | S58 AND S61 | Expanders - Apply equivalent subjects Search modes - Proximity | Interface - EBSCOhost Research Databases Search Screen - Advanced Search  Database - CINAHL Ultimate | 705 |
| S61 | S59 OR S60 | Expanders - Apply equivalent subjects Search modes - Proximity | Interface - EBSCOhost Research Databases Search Screen - Advanced Search  Database - CINAHL Ultimate | Display |
| S60 | "grit" | Expanders - Apply equivalent subjects Search modes - Proximity | Interface - EBSCOhost Research Databases Search Screen - Advanced Search  Database - CINAHL Ultimate | Display |
| S59 | "resilience" | Expanders - Apply equivalent subjects Search modes - Proximity | Interface - EBSCOhost Research Databases Search Screen - Advanced Search  Database - CINAHL Ultimate | Display |
| S58 | S42 AND S57 | Expanders - Apply equivalent subjects Search modes - Proximity | Interface - EBSCOhost Research Databases Search Screen - Advanced Search  Database - CINAHL Ultimate | Display |
| S57 | S43 OR S44 OR S45 OR S46 OR S47 OR S48 OR S49 OR S50 OR S51 OR S52 OR S53 OR S54 OR S55 OR S56 | Expanders - Apply equivalent subjects Search modes - Proximity | Interface - EBSCOhost Research Databases Search Screen - Advanced Search  Database - CINAHL Ultimate | Display |
| S56 | (MH "Golf") | Expanders - Apply equivalent subjects Search modes - Proximity | Interface - EBSCOhost Research Databases Search Screen - Advanced Search  Database - CINAHL Ultimate | Display |
| S55 | (MH "Rowing") | Expanders - Apply equivalent subjects Search modes - Proximity | Interface - EBSCOhost Research Databases Search Screen - Advanced Search  Database - CINAHL Ultimate | Display |
| S54 | "sailing" | Expanders - Apply equivalent subjects Search modes - Proximity | Interface - EBSCOhost Research Databases Search Screen - Advanced Search  Database - CINAHL Ultimate | Display |
| S53 | (MH "Aquatic Sports") | Expanders - Apply equivalent subjects Search modes - Proximity | Interface - EBSCOhost Research Databases Search Screen - Advanced Search  Database - CINAHL Ultimate | Display |
| S52 | (MH "Swimming") | Expanders - Apply equivalent subjects Search modes - Proximity | Interface - EBSCOhost Research Databases Search Screen - Advanced Search  Database - CINAHL Ultimate | Display |
| S51 | "hiking" | Expanders - Apply equivalent subjects Search modes - Proximity | Interface - EBSCOhost Research Databases Search Screen - Advanced Search  Database - CINAHL Ultimate | Display |
| S50 | (MH "Running") | Expanders - Apply equivalent subjects Search modes - Proximity | Interface - EBSCOhost Research Databases Search Screen - Advanced Search  Database - CINAHL Ultimate | Display |
| S49 | (MH "Walking") | Expanders - Apply equivalent subjects Search modes - Proximity | Interface - EBSCOhost Research Databases Search Screen - Advanced Search  Database - CINAHL Ultimate | Display |
| S48 | (MH "Wilderness Experience") OR “outdoor” | Expanders - Apply equivalent subjects Search modes - Proximity | Interface - EBSCOhost Research Databases Search Screen - Advanced Search  Database - CINAHL Ultimate | Display |
| S47 | (MH "Experiential Learning") OR "expedition" | Expanders - Apply equivalent subjects Search modes - Proximity | Interface - EBSCOhost Research Databases Search Screen - Advanced Search  Database - CINAHL Ultimate | Display |
| S46 | (MH "Wilderness Experience") OR (MH "Sports") | Expanders - Apply equivalent subjects Search modes - Proximity | Interface - EBSCOhost Research Databases Search Screen - Advanced Search  Database - CINAHL Ultimate | Display |
| S45 | "flow*" | Expanders - Apply equivalent subjects Search modes - Proximity | Interface - EBSCOhost Research Databases Search Screen - Advanced Search  Database - CINAHL Ultimate | Display |
| S44 | "nature-based" | Expanders - Apply equivalent subjects Search modes - Proximity | Interface - EBSCOhost Research Databases Search Screen - Advanced Search  Database - CINAHL Ultimate | Display |
| S43 | "natur*" | Expanders - Apply equivalent subjects Search modes - Proximity | Interface - EBSCOhost Research Databases Search Screen - Advanced Search  Database - CINAHL Ultimate | Display |
| S42 | S32 OR S33 OR S34 OR S35 OR S36 OR S37 OR S38 OR S39 OR S40 OR S41 | Expanders - Apply equivalent subjects Search modes - Proximity | Interface - EBSCOhost Research Databases Search Screen - Advanced Search  Database - CINAHL Ultimate | Display |
| S41 | "program*" | Expanders - Apply equivalent subjects Search modes - Proximity | Interface - EBSCOhost Research Databases Search Screen - Advanced Search  Database - CINAHL Ultimate | Display |
| S40 | "experience*" | Expanders - Apply equivalent subjects Search modes - Proximity | Interface - EBSCOhost Research Databases Search Screen - Advanced Search  Database - CINAHL Ultimate | Display |
| S39 | "activit*" | Expanders - Apply equivalent subjects Search modes - Proximity | Interface - EBSCOhost Research Databases Search Screen - Advanced Search  Database - CINAHL Ultimate | Display |
| S38 | "training" | Expanders - Apply equivalent subjects Search modes - Proximity | Interface - EBSCOhost Research Databases Search Screen - Advanced Search  Database - CINAHL Ultimate | Display |
| S37 | (MH "Physical Activity") | Expanders - Apply equivalent subjects Search modes - Proximity | Interface - EBSCOhost Research Databases Search Screen - Advanced Search  Database - CINAHL Ultimate | Display |
| S36 | (MH "Education") | Expanders - Apply equivalent subjects Search modes - Proximity | Interface - EBSCOhost Research Databases Search Screen - Advanced Search  Database - CINAHL Ultimate | Display |
| S35 | "sport" | Expanders - Apply equivalent subjects Search modes - Proximity | Interface - EBSCOhost Research Databases Search Screen - Advanced Search  Database - CINAHL Ultimate | Display |
| S34 | (MH "Exercise") | Expanders - Apply equivalent subjects Search modes - Proximity | Interface - EBSCOhost Research Databases Search Screen - Advanced Search  Database - CINAHL Ultimate | Display |
| S33 | (MH "Recreational Therapy") OR (MH "Recreation") | Expanders - Apply equivalent subjects Search modes - Proximity | Interface - EBSCOhost Research Databases Search Screen - Advanced Search  Database - CINAHL Ultimate | Display |
| S32 | (MH "Intervention Trials") OR (MH "Experimental Studies") | Expanders - Apply equivalent subjects Search modes - Proximity | Interface - EBSCOhost Research Databases Search Screen - Advanced Search  Database - CINAHL Ultimate | Display |
| S31 | S27 AND S30 | Expanders - Apply equivalent subjects Search modes - Proximity | Interface - EBSCOhost Research Databases Search Screen - Advanced Search  Database - CINAHL Ultimate | 986 |
| S30 | S28 OR S29 | Expanders - Apply equivalent subjects Search modes - Proximity | Interface - EBSCOhost Research Databases Search Screen - Advanced Search  Database - CINAHL Ultimate | 24,337 |
| S29 | "grit" | Expanders - Apply equivalent subjects Search modes - Proximity | Interface - EBSCOhost Research Databases Search Screen - Advanced Search  Database - CINAHL Ultimate | 739 |
| S28 | "resilience" | Expanders - Apply equivalent subjects Search modes - Proximity | Interface - EBSCOhost Research Databases Search Screen - Advanced Search  Database - CINAHL Ultimate | 23,742 |
| S27 | S11 AND S26 | Expanders - Apply equivalent subjects Search modes - Proximity | Interface - EBSCOhost Research Databases Search Screen - Advanced Search  Database - CINAHL Ultimate | 119,758 |
| S26 | S12 OR S13 OR S14 OR S15 OR S16 OR S17 OR S18 OR S19 OR S20 OR S21 OR S22 OR S23 OR S24 OR S25 | Expanders - Apply equivalent subjects Search modes - Proximity | Interface - EBSCOhost Research Databases Search Screen - Advanced Search  Database - CINAHL Ultimate | 355,436 |
| S25 | (MH "Golf") | Expanders - Apply equivalent subjects Search modes - Proximity | Interface - EBSCOhost Research Databases Search Screen - Advanced Search  Database - CINAHL Ultimate | 1,395 |
| S24 | (MH "Rowing") | Expanders - Apply equivalent subjects Search modes - Proximity | Interface - EBSCOhost Research Databases Search Screen - Advanced Search  Database - CINAHL Ultimate | 1,114 |
| S23 | "sailing" | Expanders - Apply equivalent subjects Search modes - Proximity | Interface - EBSCOhost Research Databases Search Screen - Advanced Search  Database - CINAHL Ultimate | 1,064 |
| S22 | (MH "Aquatic Sports") | Expanders - Apply equivalent subjects Search modes - Proximity | Interface - EBSCOhost Research Databases Search Screen - Advanced Search  Database - CINAHL Ultimate | 1,081 |
| S21 | (MH "Swimming") | Expanders - Apply equivalent subjects Search modes - Proximity | Interface - EBSCOhost Research Databases Search Screen - Advanced Search  Database - CINAHL Ultimate | 5,031 |
| S20 | "hiking" | Expanders - Apply equivalent subjects Search modes - Proximity | Interface - EBSCOhost Research Databases Search Screen - Advanced Search  Database - CINAHL Ultimate | 333 |
| S19 | (MH "Running") | Expanders - Apply equivalent subjects Search modes - Proximity | Interface - EBSCOhost Research Databases Search Screen - Advanced Search  Database - CINAHL Ultimate | 10,892 |
| S18 | (MH "Walking") | Expanders - Apply equivalent subjects Search modes - Proximity | Interface - EBSCOhost Research Databases Search Screen - Advanced Search  Database - CINAHL Ultimate | 26,041 |
| S17 | (MH "Wilderness Experience") OR "outdoor" | Expanders - Apply equivalent subjects Search modes - Proximity | Interface - EBSCOhost Research Databases Search Screen - Advanced Search  Database - CINAHL Ultimate | 6,063 |
| S16 | (MH "Experiential Learning") OR "expedition" | Expanders - Apply equivalent subjects Search modes - Proximity | Interface - EBSCOhost Research Databases Search Screen - Advanced Search  Database - CINAHL Ultimate | 4,289 |
| S15 | (MH "Wilderness Experience") OR (MH "Sports") | Expanders - Apply equivalent subjects Search modes - Proximity | Interface - EBSCOhost Research Databases Search Screen - Advanced Search  Database - CINAHL Ultimate | 10,494 |
| S14 | "flow*" | Expanders - Apply equivalent subjects Search modes - Proximity | Interface - EBSCOhost Research Databases Search Screen - Advanced Search  Database - CINAHL Ultimate | 107,240 |
| S13 | "nature-based" | Expanders - Apply equivalent subjects Search modes - Proximity | Interface - EBSCOhost Research Databases Search Screen - Advanced Search  Database - CINAHL Ultimate | 298 |
| S12 | "natur*" | Expanders - Apply equivalent subjects Search modes - Proximity | Interface - EBSCOhost Research Databases Search Screen - Advanced Search  Database - CINAHL Ultimate | 191,630 |
| S11 | S1 OR S2 OR S3 OR S4 OR S5 OR S6 OR S7 OR S8 OR S9 OR S10 | Expanders - Apply equivalent subjects Search modes - Proximity | Interface - EBSCOhost Research Databases Search Screen - Advanced Search  Database - CINAHL Ultimate | 1,725,300 |
| S10 | "program*" | Expanders - Apply equivalent subjects Search modes - Proximity | Interface - EBSCOhost Research Databases Search Screen - Advanced Search  Database - CINAHL Ultimate | 613,374 |
| S9 | "experience*" | Expanders - Apply equivalent subjects Search modes - Proximity | Interface - EBSCOhost Research Databases Search Screen - Advanced Search  Database - CINAHL Ultimate | 584,591 |
| S8 | "activit*" | Expanders - Apply equivalent subjects Search modes - Proximity | Interface - EBSCOhost Research Databases Search Screen - Advanced Search  Database - CINAHL Ultimate | 482,021 |
| S7 | "training" | Expanders - Apply equivalent subjects Search modes - Proximity | Interface - EBSCOhost Research Databases Search Screen - Advanced Search  Database - CINAHL Ultimate | 286,385 |
| S6 | (MH "Physical Activity") | Expanders - Apply equivalent subjects Search modes - Proximity | Interface - EBSCOhost Research Databases Search Screen - Advanced Search  Database - CINAHL Ultimate | 56,211 |
| S5 | (MH "Education") | Expanders - Apply equivalent subjects Search modes - Proximity | Interface - EBSCOhost Research Databases Search Screen - Advanced Search  Database - CINAHL Ultimate | 11,552 |
| S4 | "sport" | Expanders - Apply equivalent subjects Search modes - Proximity | Interface - EBSCOhost Research Databases Search Screen - Advanced Search  Database - CINAHL Ultimate | 29,363 |
| S3 | (MH "Exercise") | Expanders - Apply equivalent subjects Search modes - Proximity | Interface - EBSCOhost Research Databases Search Screen - Advanced Search  Database - CINAHL Ultimate | 61,834 |
| S2 | (MH "Recreational Therapy") OR (MH "Recreation") | Expanders - Apply equivalent subjects Search modes - Proximity | Interface - EBSCOhost Research Databases Search Screen - Advanced Search  Database - CINAHL Ultimate | 6,932 |
| S1 | (MH "Intervention Trials") OR (MH "Experimental Studies") | Expanders - Apply equivalent subjects Search modes - Proximity | Interface - EBSCOhost Research Databases Search Screen - Advanced Search  Database - CINAHL Ultimate | 37,780 |

eTable 10. Web of Science Search Results

| 1 | Intervention* (Topic) OR recreation* (All Fields) OR exercise* (All Fields) OR sport* (All Fields) OR education* (All Fields) OR physical* (All Fields) OR physical education and training (All Fields) OR therap* (All Fields) OR training* (All Fields) OR activit* (All Fields) OR experience* (All Fields) OR program* (All Fields)  Date Run: Tue Nov 26 2024 08:25:54 GMT+0800 (China Standard Time) | 28321109 |
| --- | --- | --- |
| 2 | natur* (Topic) OR nature-base* (All Fields) OR flow* (All Fields) OR adventure* (All Fields) OR expedition* (All Fields) OR experiential* (All Fields) OR outdoor* (All Fields) OR wilderness* (All Fields) OR walking (All Fields) OR running (All Fields) OR hiking (All Fields) OR swimming (All Fields) OR surf* (All Fields) OR water sports (All Fields) OR sailing (All Fields) OR sail* (All Fields) OR kayak* (All Fields) OR rowing (All Fields) OR golf* (All Fields)    Date Run: Tue Nov 26 2024 08:31:12 GMT+0800 (China Standard Time) | 13124411 |
| 3 | #1 AND #2  Date Run: Tue Nov 26 2024 08:31:50 GMT+0800 (China Standard Time) | 5530348 |
| 4 | ALL=(psychological resilience)  Date Run: Tue Nov 26 2024 08:32:41 GMT+0800 (China Standard Time) | 19208 |
| 5 | #4 AND #3  Date Run: Tue Nov 26 2024 08:32:55 GMT+0800 (China Standard Time) | 1630 |

eTable 11. Comprehensive Details of the GRADE Tool


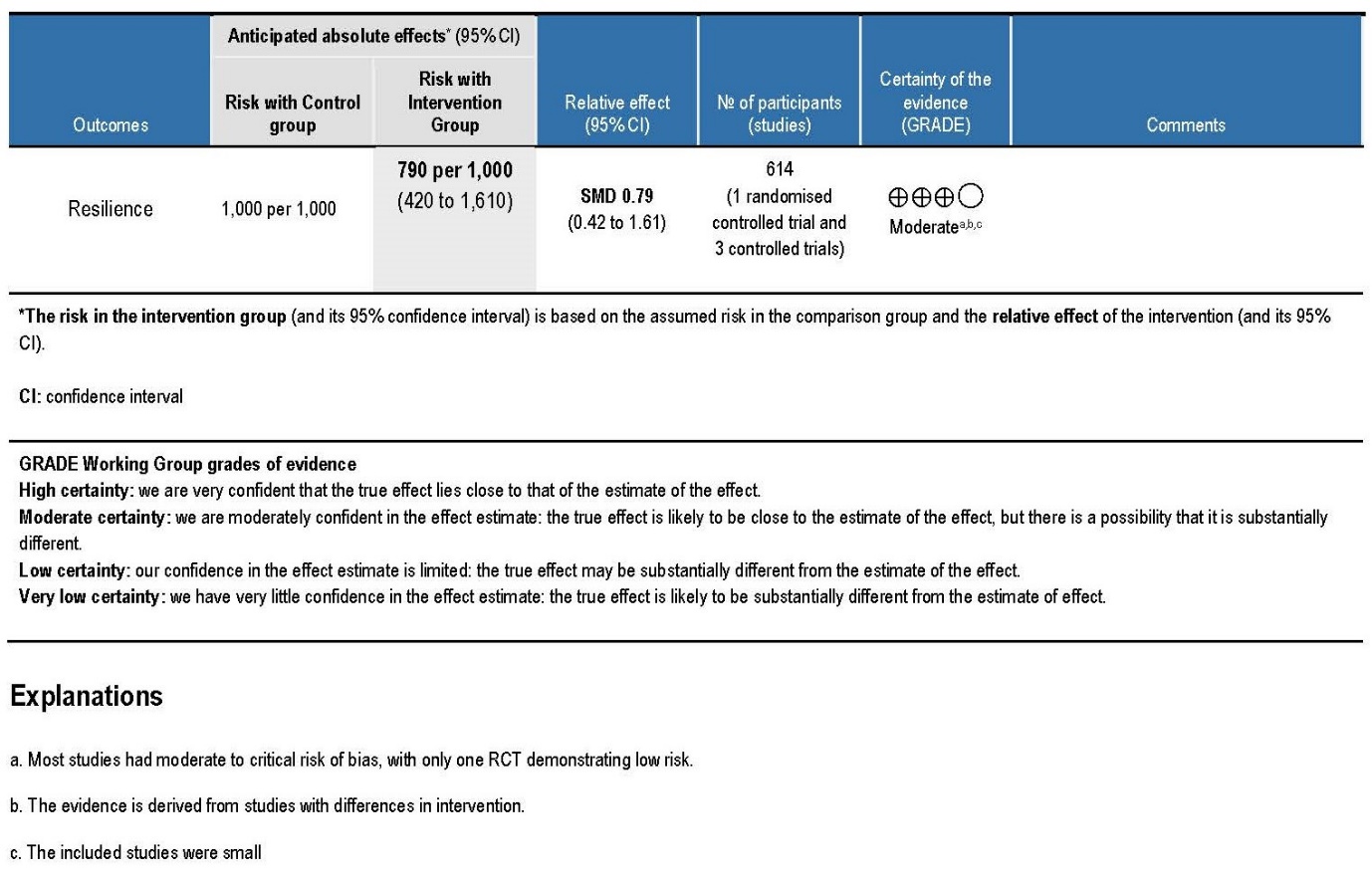


eFigure 1: Funnel Plot for Assessing Publication Bias


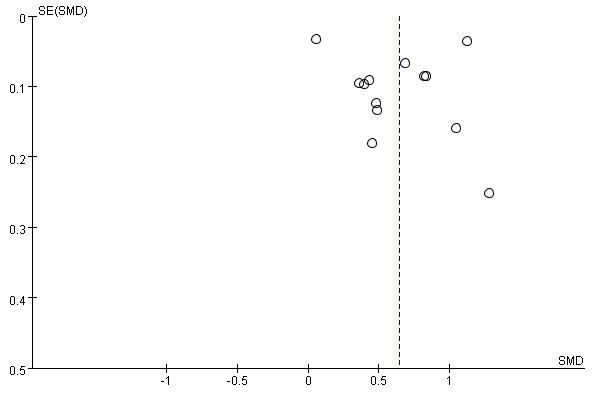


eTable 12. Summary Description of the Characteristics of Included Studies

| Authors | Country | Study design | Sample size | Ages (Mean ± SD), Range | % of female | Attrition rate |
| --- | --- | --- | --- | --- | --- | --- |
| Albedry et al., 2023 | USA | PPS | 95 | 15 ± .56 | 52.6 | 6.32% |
| Allan et al., 2024 | UK | PPS | 622 | 16-17 years | 58 | 48.4% |
| Arahanga-Doyle et al., 2019 | New Zealand | PPS | 91 | 15.25 | 60.4 | nd |
| Boarini et al., 2024 | Italy | PPS | 25 | Median of 16 | 52 | nd |
| Bowen et al., 2016 | Australia | PPS | 36 | 14.6 ± 1.6 | 58.3 | nd |
| Chung et al., 2021b | Hong Kong China | RCT | 228 | 13 ± 0.8 | 45.2 | 0 |
| Furness et al., 2017 | New Zealand | CT | 80 | 13 to 15 years | 41.3 | 4% |
| Gillespie & Allen-Craig, 2009 | Australia | PPS | 19 | 14 to 17 years | 0 | 17.4% |
| Hayhurst et al., 2015_Study 1 | New Zealand | CT | 126 | 16.58 | 57.1 | nd |
| Hayhurst et al., 2015_Study 2 | New Zealand | PPS | 146 | 15.25 | 50 | nd |
| Katisi et al., 2019 | Botswana | PPS | 650 | 13.42 ± 1.03 | 52.6 | nd |
| Koni et al., 2019_Study 1 | New Zealand | PPS | 136 | 16.58 ± 1.73 | 60.3 | nd |
| Koni et al., 2019_Study 2 | New Zealand | PPS | 91 | 15.25 ± 1.18 | 60.4 | nd |
| Ritchie et al., 2014 | Canada | PPS | 46 | 14.6 | 17.4 | 20.3% |
| Scarf et al., 2017 | New Zealand | CT | 180 | 16.54 | 56.7 | nd |

^1^ RCT: Randomized controlled trial; CT: Control trial; PPS: Pre-post study; ² nd: No data

eFigure 2. Revised Cochrane Risk-of-Bias tool for randomized trials (RoB 2) Summary and Author Judgments of Low, Some concerns, and High Risk of Bias Across All Included RCTs studies (N = 1)


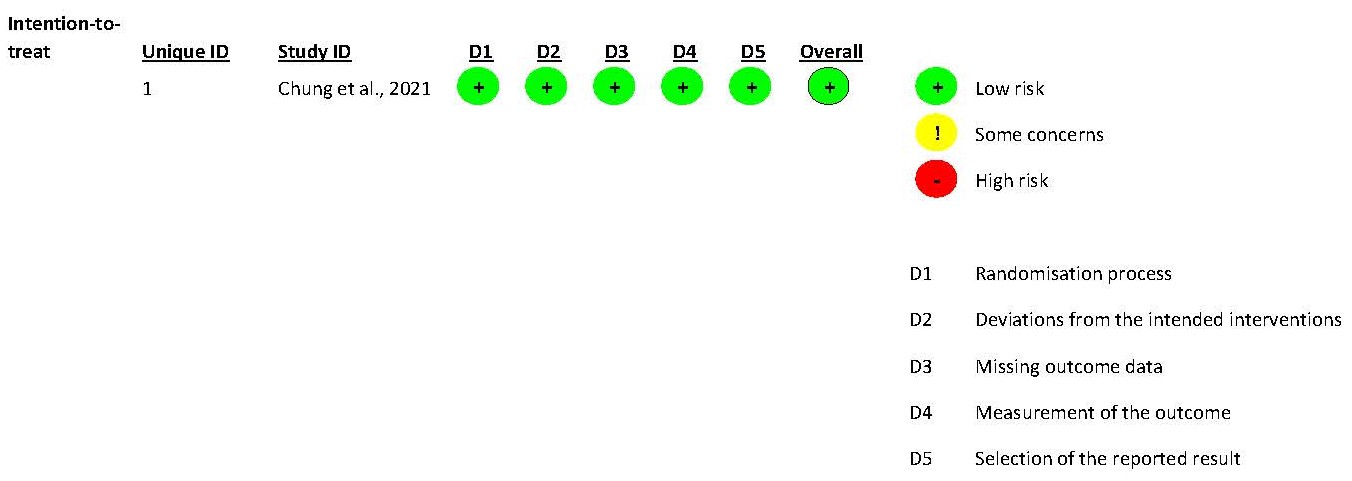


eFigure 3. Cochrane Risk of Bias in Non-randomized Studies – of Intervention (ROBINS-I) Summary and Author Judgments of Low, Moderate, serious, and Critical Risk of Bias Across All Included Non-RCTs Studies (N = 14)


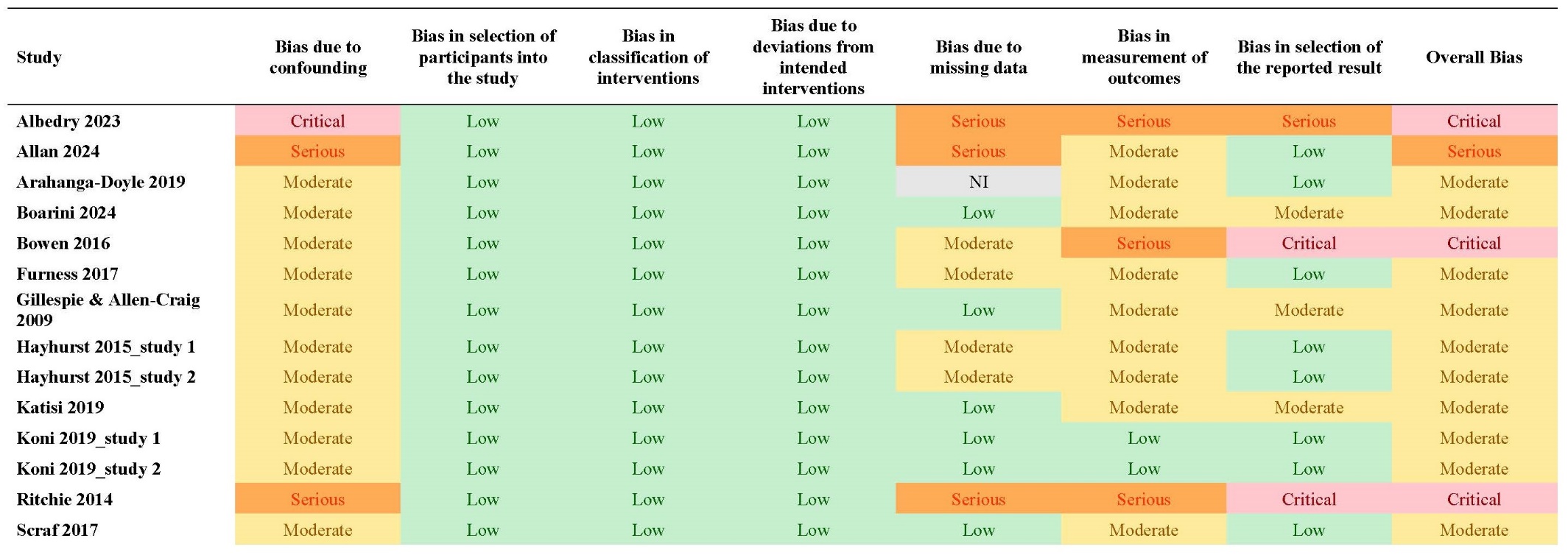


eTable 13. Summary Description of the Outcomes and Measurements of Included Studies

| Study | Design | Resilience needs/ Challenges of targeted participants | Evidence of resilience | Assessment timepoint | Outcome measures | Funding | Socioeconomic status |
| --- | --- | --- | --- | --- | --- | --- | --- |
| Albedry et al., 2023 | PPS | Decreased motivation & engagement; disengagement from school; high-risk behaviours; decline in social-emotional learning competencies | Yes  CD-RISC (t = 5.686, p < .001) | Pre and post | Resilience: CD-RISC  Physical activity: IPAQ | nd | No |
| Allan et al., 2024 | PPS | Physical inactivity; mental health problems; social disconnection; educational disparities; special educational needs and disabilities | Yes  Pre-post: CD-RISC (Cohen’s d =0.58)  FU: Significant increase retained | Pre, post and one-month FU | Resilience: CD-RISC  Psychological well-being: SWEMWBS | nd | No |
| Arahanga-Doyle et al., 2019 | PPS | Mental health issues; socioeconomic disadvantages; cultural alienation; poor Sense of Identity | Yes  Māori youth: (t = 7.412, p < .001)  New Zealand European youth: (t = 4.202, p < .001) | Pre and post | Resilience: RS  Self-esteem: Self-Esteem Scale  Positive outlook: Self-Esteem Scale  Social identity: Ellmers’s self-categorization subscale  Social Support: Neill and Dias’ Social Support scale | nd | Yes |
| Boarini et al., 2024 | PPS | Physical challenges; anxiety; depression; low self-esteem; feelings of helplessness & hopelessness; social challenges; isolation | Yes  Narratives | Pre and post | Resilience: BESSI-20  QoL: EQ-5D  Behavioural, Emotional, and Social Skills: BESSI-20 | nd | Yes |
| Bowen et al., 2016 | PPS | Mental health issues; social and cultural changes; learning problems;  disengagement from education; family Issues; delinquency and substance abuse | Yes  Pre-post: RQ (Cohen’s d = 0.49)  FU: Insignificant slight reduction | Pre, post and three-month FU | Resilience: RQ  Depressive symptoms: BDI-II  Behavioural and emotional functioning: YSR  Self-esteem:CSEI  Family functioning: CORE FFQ  Suicidal proneness: LAS-SF | nd | No |
| Chung et al., 2021b | RCT | Mental health issues; academic & parental pressure; developmental changes; poverty: | Yes  ES (Partial η2)  Pre-post: RS-14 (Medium ES=0.06)  FU: RS-14 (Small to moderate ES=0.05) | Pre, post and six-month FU | Resilience: RS  Depressive symptoms: CES‐DC  Self-esteem: RSES | Yes | Yes |
| Furness et al., 2017 | CT | Low self-efficacy; risky behaviours; mental health issues; social & emotional changes | Yes  RS (Cohen’s d =0.7) | Pre and post | Resilience: RS  Self-efficacy: PKSEQ  Connectedness: HACS  Psychological wellbeing: The Affectometer 2 | nd | Yes |
| Gillespie & Allen-Craig, 2009 | PPS | Behavioural problems; school-related issues; mental health issues; being victims of bullying; substance abuse | Yes  Narratives | Pre, during, and post | Resilience: RS  Protective factors: YARPET | nd | No |
| Hayhurst et al., 2015_Study 1 | CT | Depression, suicide; poverty; mental health issues; lack of social competence | Yes  RS (t = 5.54, p < 0.0005, η2 = 0.35) | Pre and post | Resilience: RS | nd | No |
| Hayhurst et al., 2015_Study 2 | PPS |  | Yes  Pre-post: RS (t = 4.18, p < 0.0005, η2 = 0.20)  FU: Not significant | Pre, post and five-month FU | Resilience: RS  Social effectiveness: ROPE  Self-efficacy: ROPE  Self-esteem: The Self-Description  Questionnaire III  Belonging: Sheldon and Bettencourt’s three-item inclusion  scale  Social support: Neill and Dias (2001) 4-item scale  Perception of weather: |  |  |
| Katisi et al., 2019 | PPS | Grief & loss; poverty; exploitation & abuse; social disruption; stigma & discrimination; educational challenges; mental health issues | Yes  Males: Cohen’s d = 0.14  Females: Cohen’s d = 0.10  At-risk males: Cohen’s d = 0.42  At-risk females: Cohen’s d = 0.31 | Pre and post | Resilience: CYRM-28  Impact of Grief: Inventory of Complicated Grief  Future aspiration: Aspirations/Peer Leader Survey | Yes | No |
| Koni et al., 2019_Study 1 | PPS | Low resilience; socioeconomic challenges; mental health disparities; identity problem; developmental challenges; social isolation | Yes  RS (Hedge’s g 0.69; 95% CI, 0.44 to 0.93, p<.001) | Pre and post | Resilience: RS  Social identity: Sheldon and Bettencourt’s 3-item inclusion scale | nd | No |
| Koni et al., 2019_Study 2 | PPS |  | Yes  RS (Hedge’s g 0.83; 95% CI: 0.52 to 1.31, p>.001) | Pre and post |  |  |  |
| Ritchie et al., 2014 | PPS | Lower health status; difficult living conditions; cultural Identity issues; hopelessness; high suicidal & mental illness rates; family instability; life stressors: | Yes  Pre-post: RS (Hedge’s g= 0.36; t=2.64, p=.011)  FU: Not significant | Pre, post and 12-month FU | Resilience: RS  Change in resilience: MCS | Yes | No |
| Scarf et al., 2017 | CT | Mental health problems; social Isolation | Yes  Significant main effect of Time on resilience scores, F (1, 178) = 49.43, p < .001, η² = .22  Pre-post  IG: RS (t= 8.40, p < .0005, η² = .44)  CG: Not significant | Pre and post | Resilience: RS  Perceived social support: Neill and Dias 4-item scale  Sense of belonging: Sheldon and  Bettencourt’s 3-item group inclusion scale | No | No |

BDI-II: The Beck Depression Inventory-II

BESSI-20: The Behavioral, Emotional, and Social Skills Inventory – Observer report form

CD-RISC: The Connor-Davidson Resilience Scale

CES‐DC: The Center for Epidemiologic Studies Depression Scale for Children

CORE FFQ: The CORE Family Functioning Questionnaire

CSEI: The Coopersmith Self Esteem Inventory

CYRM-28: The Child and Youth Resilience Measure

HACS: Hemingway Adolescent Connectedness Scale

IPAQ: The International Physical Activity Questionnaire

LAS-SF: The Life Attitudes Schedule – Short Form

MCS: The Mental Component Score

PKSEQ: The revised Project K Self-efficacy Questionnaire

ROPE: The Review of Personal Effectiveness

RQ: Resilience questionnaire

RS: The Wagnild and Young’s (1993) Resilience scale

RSES: The Rosenberg's Self‐Esteem Scale

SWEMWBS: The Shortened Warwick—Edinburgh Mental Wellbeing Scale

YARPET: The Life Effectiveness Questionnaire - Youth at-risk version

YSR: The Youth Self-Report

eFigure 4. Sensitivity Analysis Using a Correlation of 0.5


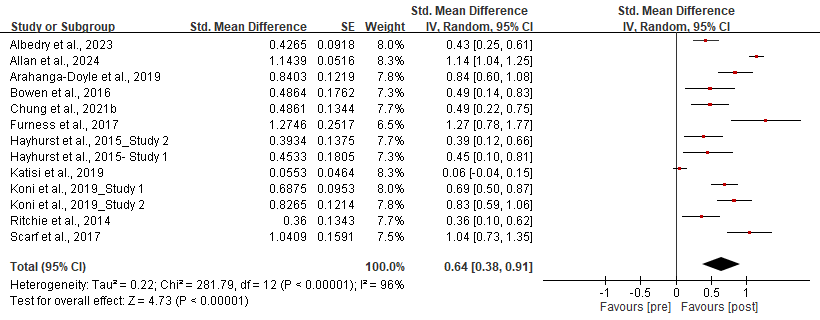


eFigure 5. Sensitivity Analysis Excluding Single-Group Pre-Post Studies
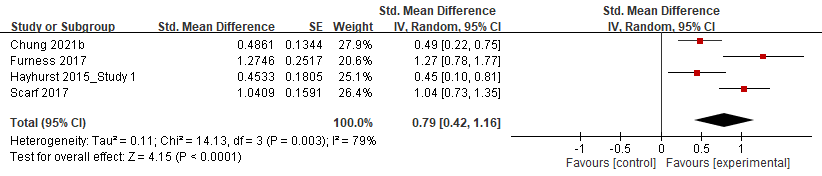


eFigure 6. Sensitivity Analysis Excluding Studies with Serious or Critical Risk of Bias


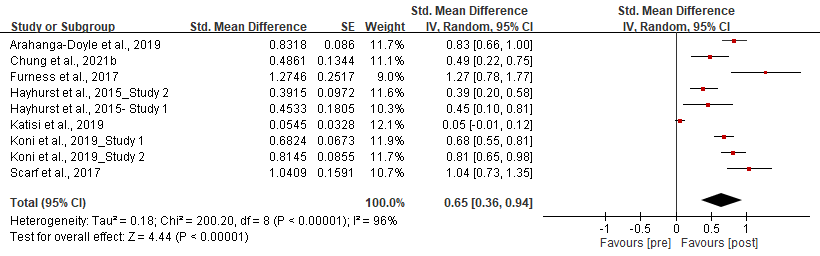


eFigure 7. Pooled Effect Sizes for Within-Group Pre–Post Changes in Intervention Arms of Controlled Trials and Single-Group Pre–Post Studies


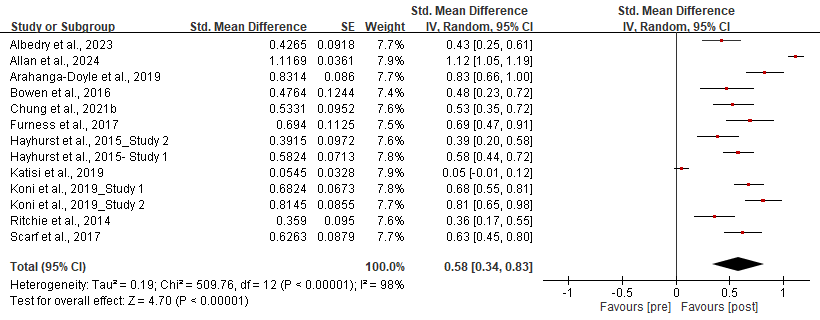

Supplement: Supplementary file 1 — Supplementary file1 (DOCX 992 kb) [file 44192_2025_258_MOESM1_ESM.docx]
